# Multi-omics integration of transcriptome, miRNA, and metabolome uncovers molecular mechanisms of male flower development in cucumber line B10 (*Cucumis sativus* L.)

**DOI:** 10.1038/s41598-025-28485-6

**Published:** 2025-11-29

**Authors:** Szymon Turek, Agnieszka Skarzyńska-Łyżwa, Aparna Aparna, Wojciech Pląder, David Riewe, Astrid Junker, Thomas Altmann, Magdalena Pawełkowicz

**Affiliations:** 1 Department of Plant Genetics, Breeding and Biotechnology, Institute of Biology, Warsaw University of Life Sciences, Nowoursynowska 159, Warsaw, 02-776 Poland; 2 Leibniz Institute of Plant Genetics and Crop Plant Research (IPK), Corrensstrasse 3, 06466 Seeland, OT Gatersleben Germany; 3 Institute for Ecological Chemistry, Plant Analysis and Stored Product Protection, Julius Kuehn Institute (JKI) - Federal Research Centre for Cultivated Plants, Koenigin-Luise-Strasse 19, 14195 Berlin, Germany; 4 Syngenta Seeds GmbH, Zum Knippenbach 20, 32107 Bad Salzuflen, Germany

**Keywords:** Floral organogenesis, Male flower development, Cucumber (*Cucumis sativus*), Multi-omics integration, Regulatory networks, Reproductive biology, miRNA-target interactions, Metabolite profiling, Plant reproduction, Plant molecular biology, Plant development, Gene regulatory networks, High-throughput screening

## Abstract

Male flower development in cucumber (*Cucumis sativus* L.) is a highly coordinated and genetically regulated process, yet the full complexity of its molecular underpinnings remains incompletely understood. In this study, we present a comprehensive, multi-omics analysis of male flower development in the cucumber line B10, integrating transcriptomic (RNA-seq), small RNA (miRNA) profiling, and metabolomic data across key tissues, including leaves, shoot apex, and floral buds at distinct developmental stages. Our analyses reveal dynamic gene expression changes and novel regulatory miRNAs, several of which have not previously been linked to male bud formation in cucumber. Functional enrichment analyses using GO and KEGG highlight critical pathways, including starch and sucrose metabolism, carbohydrate utilization, sporopollenin biosynthesis, and lignin catabolism. An integrative analysis combining miRNA–target interactions, transcriptomic shifts, and differential metabolite accumulation revealed coherent regulatory cascades linking transcription factors, carbohydrate metabolism, and cell wall dynamics. This study provides novel insights into the intricate genetic and metabolic networks shaping male flower morphogenesis and provides a valuable resource for advancing cucumber reproductive biology and crop improvement strategies.

**Supplementary Information:**

The online version contains supplementary material available at 10.1038/s41598-025-28485-6.

## Introduction

Cucumber, an important vegetable worldwide, exhibits a complex mechanism of sexual differentiation that directly affects yield. Despite considerable progress, the molecular basis of sex determination in cucumber remains incompletely understood, reflecting the multifactorial nature of this process. Cucumber exhibits a variety of plant sex forms due to the presence of three distinct types of flowers: staminate (male), pistillate (female), and hermaphroditic. These flower types can be combined in multiple configurations, resulting in different plant sex phenotypes^1^. This process has been shown to be influenced by genetic, hormonal and environmental factors^1–4^. In particular, ethylene and auxins play key role in promoting female flower development, while other hormones like gibberellins can promote male flower formation^5^. Mutations causing ethylene insensitivity can prevent the transition to female flowers, resulting in the continued production of male flowers^6^. Therefore, sex differentiation in cucumber is a multifactorial phenomenon, driven by the interaction of different signals and conditions that affect the reproductive success and yield of the plant^2,7^.

During the growth of the flower buds, we can distinguish specific growth phases where we can see the changes in the phenotype of the flower buds. Immediately after their formation, flower buds do not differ phenotypically and at the stage of 1–2 mm have primordia of male and female organs. At the next stage of development - at the stage of 3–5 mm - the bud is already sexually differentiated^8^. At the following stages, the sex-determined flower buds continue to grow. Thus, the earliest phases (1–2 mm and 3–5 mm) are particularly important for understanding sex determination in cucumber.

While female flower development has been extensively studied due to its direct relevance for fruit production, the regulation of male flower development remains less well understood, even though it is equally critical for viable pollen supply and successful fertilization. On the other hand, male flowers play a vital role in the pollination process. Without properly functioning male flowers, there would be no viable pollen to fertilize female flowers, which is necessary for fruit set in most cucumber varieties. Male flower development affects pollen production and quality, which in turn impacts the success of pollination, the quality of the fruit, and overall yield. Moreover, understanding male flower development can help address issues like male sterility, which can limit reproduction and crop productivity^9^.

The cucumber line under investigation is the B10 line, the highly inbred cucumber (*Cucumis sativus*) line, which is a central European male Borszczagowski line with male flowers up to the sixth node. This line also has an available reference genome^10,11^, facilitating genomic studies utilizing next-generation sequencing.

**Fig. 1:**
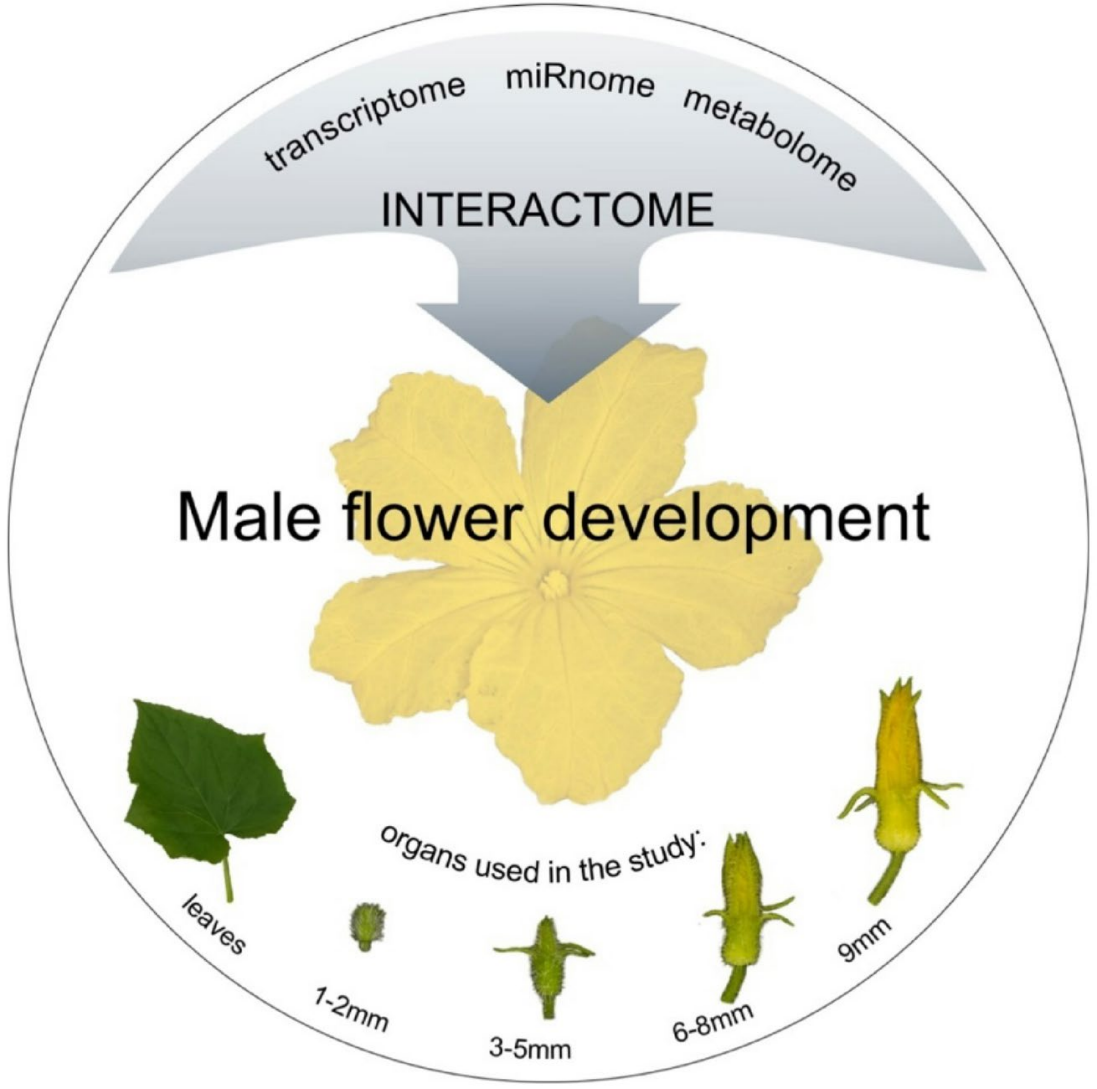
An infographic showing the main objectives of the study.

In this study, we performed a multi-omics analysis of the B10 cucumber line, integrating small RNA, transcriptomic, and metabolomic data to explore the molecular mechanisms underlying male flower development (Fig. 1). By linking miRNA–target networks with transcriptional and metabolic profiles, we identified conserved and novel regulatory modules and proposed a framework that explains how molecular layers interact to control male flower morphogenesis. This knowledge is essential for improving cucumber breeding programs aimed at enhancing both yield and quality.

## Materials and methods

**Fig. 2:**
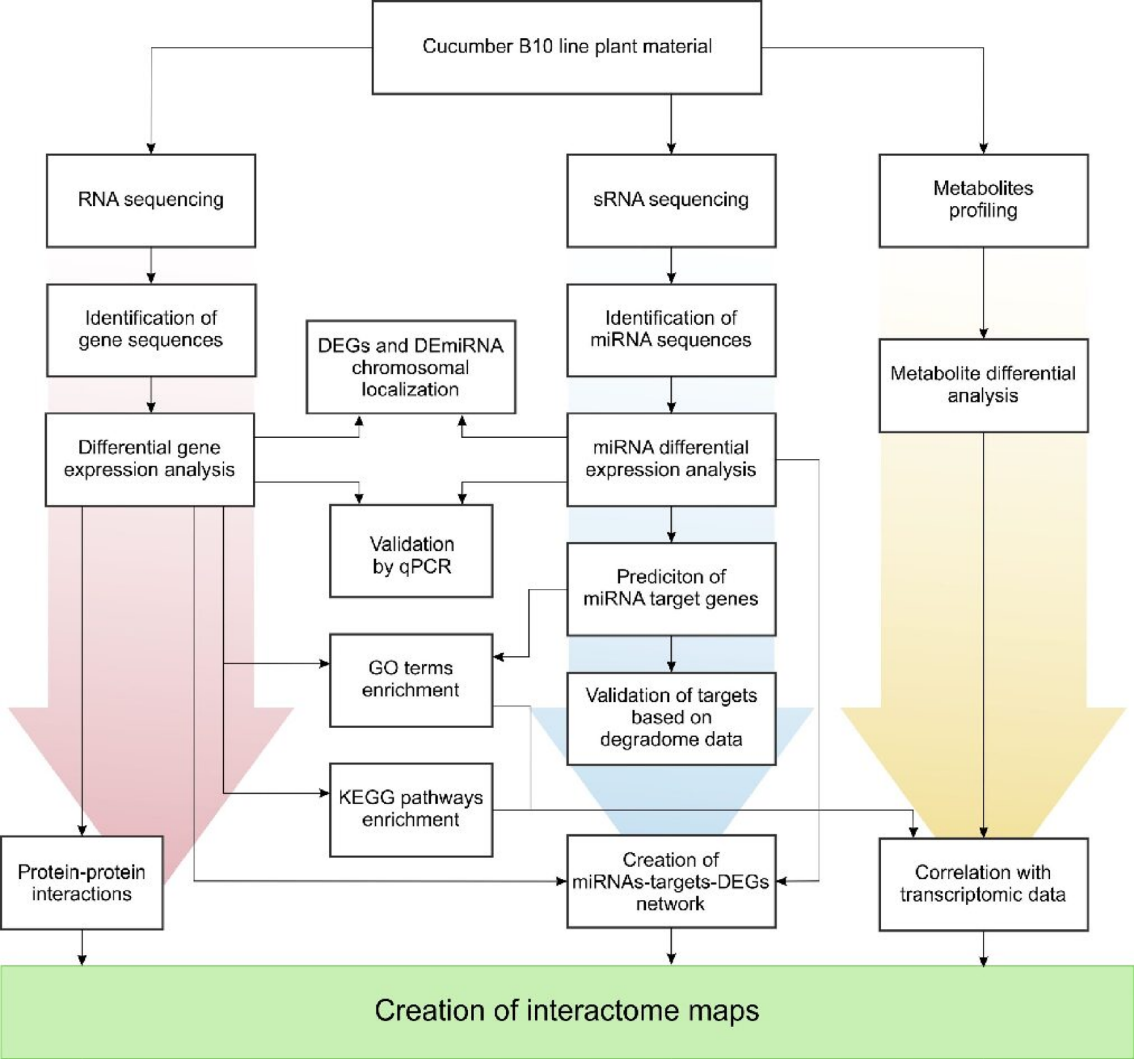
Diagram showing the workflows of the analysis carried out.

In this study, we analysed the cucumber line B10, which is a highly inbred, monoecious type of the ‘Borszczagowski’ variety, the seeds of which came from the collection of the Genetics Department of Plant Genetics, Breeding and Biotechnology of the Warsaw University of Life Sciences. In the context of our previous studies on genome sequencing and organisation^4,10^, the analyses carried out on leaves, shoot apices with small flower buds and subsequent developmental stages of male flower buds were at different molecular levels: mRNA, sRNA and metabolites. The course of the analysis is shown in Fig. 2.

### Plant material and cultivation

Plants were grown in a greenhouse under a 16 h light (25–27 °C)/8 h dark (18–20 °C) photoperiod. The light intensity (i.e., photosynthetically active radiation) in the greenhouse was provided with Philips SON-T AGRO 400 sodium lamps and an average 1500 µmol m^−2^ s^−1^. For a subsequent analysis leaves (L), shoot apex (SA - with newly formed, 1 mm long floral buds), and male floral buds up to the 6th node at four developmental stages: 1–2 mm (SI), 3–5 mm (SII), 6–8 mm (SIII) and 9 mm (SIV) were collected, frozen in liquid nitrogen, and stored at − 80 °C. For sequencing and real-time quantitative PCR (RT-qPCR) validation, samples were pooled, with each pool comprising material from 15 plants (*n* = 15), and three biological replicates were prepared, and eight pools were prepared for metabolomic profiling. All plant materials used in this study (*Cucumis sativus* L. line B10) were obtained in compliance with relevant institutional, national, and international guidelines and legislation, and were cultivated in the greenhouse at the Wolica Experimental Station, Department of Plant Genetics, Breeding and Biotechnology, Warsaw University of Life Sciences (WULS, Warsaw, Poland). In all experiments, leaves corresponded to fully expanded, mature (non‑senescent) laminae harvested from the mid‑canopy (nodes 4–6), i.e., the first fully expanded leaf below the shoot apex on the same plants used for buds collections.

### Isolation of RNA

Total RNA was extracted using the RNeasy Mini Kit (Qiagen, Valencia, CA, USA), with an additional step of DNase I treatment, according to the manufacturer’s protocol. The nucleic acid concentration and quality were assessed using a NanoDrop 2000 spectrophotometer (Thermo Fisher Scientific, Waltham, MA, USA). For RNA quality assessment, the RNA integrity number (RIN) was calculated using a Bioanalyzer 2100 (RNA 6000 Nano Kit) according to the manufacturer’s instructions (Agilent, Palo Alto, CA, USA). The RNA samples for sequencing contained total RNA ≥ 20 µg, RNA concentration ≥ 500 ng/µl, major ribosomal subunit ratio 28 S:18 S ≥ 1, and RIN ≥ 8.

### Library construction and deep sequencing

Approximately 5–10 µg of total RNA was used to construct each RNA-seq and sRNA-seq library. The RNA samples used for the sRNA-seq were the same as those used for the mRNA-seq. Polyadenylated RNA purification, RNA fragmentation (with the average 300 bp), cDNA synthesis, and PCR amplification were performed according to the Illumina RNA-seq protocol (Illumina, Inc., San Diego, CA, USA). The TrueSeq Small RNA library kit (Illumina, Inc.) was used for the sRNA library. Parallel sequencing was performed on an Illumina HiSeq 2000 platform (McGill University Genome Quebec Innovation Centre, Montreal, QC, Canada). We obtained 100-bp paired-end sequence reads for RNA-seq and 50-bp single-end sequenced reads for sRNA-seq. Read quality was assessed using the Illumina purity filter, percent low quality reads, and distribution of phred-like scores at each cycle. All the presented data passed the quality control filtering on the basis of these metrics. FastQC^12^ was used to assess the quality of the short reads. The sequences generated in this study have been deposited in the Sequence Read Archive (SRA) at the National Center for Biotechnology Information under the accession numbers BioProjects: PRJNA1166086.

### RNA-seq data analysis

To investigate gene expression and identify differentially expressed genes (DEGs), the cucumber genome sequence B10v3 (GenBank: LKUO00000000) served as the reference genome^10^. Gene expression values were estimated using Salmon^13^ with sequence-specific and GC content bias enabled. Differential expression analysis was performed using the Limma package^14^. Genes with a logarithm of fold change greater than 1.5 and a false discovery rate (FDR) less than 0.001 were selected as differentialy expressed genes (DEGs). To assess the reproducibility and repeatability of the probes and to cluster genes we used gplots library, while principal component analysis was performed using the R package with the ‘ggplot2’ package. To visualize the DEGs position across chromosome maps the R package “circlize”^15^ was applied.

### Gene ontology analysis

To evaluate the functional implications of DEGs involved in the developmental process of cucumber B10 line, we performed a Gene Ontology (GO) analysis using the topGO package^16^ for R. This analysis included the categorization of genes into biological processes, molecular functions, and cellular components. The gene dataset under investigation was derived from RNA-seq results, and we applied a significance threshold of 0.05 for the FDR and a cutoff of ± 1.5 log_2_-fold change to identify DEGs. The chosen statistical test included the classic Fisher’s exact test and the elimination algorithm for constructing the GO graph. Gene visualization was presented using topGO library - dot plot graphs and a hierarchical GO graph to illustrate the hierarchical relationships between enriched GO terms.

### KEGG analysis

In our study, KEGG^17–19^ enrichment analysis was performed using the same set of genes used in the Gene Ontology (GO) analysis, which consisted of genes that met a significance threshold of 0.05 for the FDR and exhibited a ± 1.5 log_2_-fold change. The KEGG enrichment analysis was performed using the clusterProfiler package^20^ in the R programming language, and dot plots were generated to visually represent the results. The enrichKEGG function was applied with specific parameters, including a p-value cutoff of 0.05, the Benjamini-Hochberg (BH) method for p-value adjustment, and a q-value cutoff of 0.05.

### Identification and profiling of cucumber miRNAs

The resulting sequencing files were pre-processed using fastp software^21^ which filtered and trimmed the reads to a minimum length of 15 nt. The genome of *Cucumis sativus* B10^10^ was used as a reference for the mapping of individual samples. The programs miRDeep2^22^ and ShortStack^23^ were used to detect mature miRNAs in the sRNA sequencing data. For the miRDeep2 analysis, mature miRNA sequences were downloaded from the PmiREN^24^ database together with the hairpin sequences. Due to the small amount of data for *Cucumis sativus*, the reference data was extended with data for *Cucumis melo* and *Arabidopsis thaliana*.

Based on the extracted count matrix for mature miRNAs from the miRDeep2 program, differentially expressed miRNAs (DEmiRNAs) were identified using the EdgeR package^25^, using the quasi-likelihood ratio statistical test and the standard FDR = 0.05 cutoff threshold. The sRNA sequences were detected using the *de-novo* module of the ShortStack program, from the results of which counting matrices were created based on the detected sRNA sequences classified into Y and N15 categories. The differential miRNA expression analysis was then performed in the edgeR program. In the next step, the targets to which differentially expressed miRNA molecules bind were found using the psRNATarget server^26^ and the reference transcriptome of *Cucumis sativus* B10. The found transcripts were annotated based on the annotation available for the B10 reference genome made in the publication and the annotation available in the CuGenDBv2 database^27^. The annotated transcripts were then searched for in the results of the RNA-seq analysis. Importantly, the predicted interactions were cross-validated with degradome sequencing data (Cleaveland4), which provided experimental evidence of transcript cleavage at miRNA binding sites.

### Degradome data analysis

The degradome (collection of RNA fragments that result from the cleavage of mRNA transcripts) was analyzed using the Cleaveland4 program^28^. As a reference, we used publicly available fasta files from the SRA database, specifically from the *Cucumis sativus* degradome, with identifiers SRR10198466 (derived from leaf tissue sequencing) and SRR7620953 (derived from shoot apical tissue sequencing). The input data included in silico-identified miRNA sequences based on differential miRNA expression analysis, alongside the reference transcriptome for the cucumber line B10. The software was executed with a significance parameter (p-value) set at 0.05, yielding outcomes for all four predefined categories by which the program classifies the identified results. Subsequently, target plots were generated to illustrate the locations of transcript cleavage facilitated by the selected miRNA molecules. This degradome-based validation confirms that the interactions predicted by psRNATarget are not limited to in silico predictions but are supported by experimental cleavage evidence.

### Validation of expression profiles of DEGs and DEmiRNAs by qPCR

Total RNA was isolated using the miRNeasy Mini Kit (Qiagen) according to the standard protocol, effective for obtaining both mRNA and miRNA. Synthesis of cDNA was conducted using the High Capacity cDNA Reverse Transcription kit (Thermo Fisher Scientific) with random primers for mRNA analysis and specific RT primers for miRNA analysis. Primers were designed using Primer3 (version 2.3.6) (Table Supplement 1). All qPCR assays were performed with three technical and three biological replicates.

The validation of DEGs was performed for 12 randomly selected genes. As a reference *CYCL* and *CACS* genes were used^29^. The qPCR assays were completed with the Power SYBR^®^ Green PCR Master Mix (Thermo Fisher Scientific) starting with 0.2 µg of cDNA. The qPCR program was 50 °C for 2 min, 95 °C for 10 min, followed by 40 cycles of 15 s at 95 °C and 1 min at 60 °C, using an Applied Biosystems 7500 Real-Time PCR System (Thermo Fisher Scientific). Melting curve analysis was performed immediately after the qPCR. Amplification efficiency was assessed using LinRegPCR (version 2015.3)^30^ and was 65%-80% for all primers. Relative expression levels were determined using the 2 ^− ΔΔCt^ method from the EasyqpcR-package^31^.

The miRNA expression validation was performed for 6 different miRNAs by two-tailed RT-qPCR^32^. The qPCR assays were completed with PerfeCTa SYBR Green FastMix (Quantabio) starting with 0.2 µg of cDNA. The qPCR program was 95 °C for 1 min, followed by 45 amplification cycles consisting of 95 °C for 10 s and 60 °C for 20 s, followed by melt curve analysis. Relative expression levels were determined using the 2^−ΔΔCt^ method. As the reference for miRNA analysis U6 gene was used.

### GC-MS analysis for metabolite profiling

Metabolite extraction, in-line derivatization and GC-MS analysis were carried out as described previously^33–36^. Fifteen mg of deep-frozen and ground sample material was used for extraction. The extracts were dried in GC-MS glass-vials in a RVC 2–33 CDplus vacuum concentrator (Martin Christ, Osterode, Germany). They were derivatized in-line directly prior to each injection using an MPS2-XL autosampler (Gerstel, Muehlheim, Germany). 1 µl was splitless injected and analysed in an Agilent 7890 gas chromatography (Agilent, Santa Clara, CA, USA) coupled to a Pegasus HT mass spectrometer (LECO, St. Joseph, MI, USA). Known and unknown metabolites were identified and assigned using LECO Chroma TOF software including the Statistical Compare package and the mass spectral library provided by the Golm Metabolome Database (GMD, http://gmd.mpimp-golm.mpg.de/). Relative abundancies of known and unknown metabolites were extracted from the chromatograms using the r-package TargetSearch^37^.

For the statistical analysis, the MetaboDiff package^38^, was used. Principal Component Analysis (PCA) and analysis of differentially accumulated metabolites (DAMs) was performed with the Student’s t-test, and p-value correction using the Benjamini-Hochberg procedure. Metabolites with adjusted p-values below 0.05 were considered as significantly differentiating accumulated metabolites (DAMs).

### Creation of interactome maps

In silico analysis of protein-protein interaction of DEGs was performed using STRING software^39^. One thousand nine hundred ninety-eight DEGs with log_2_FC > 3 were selected for analysis. The proteins forming the network were divided into 6 clusters using k-means clustering. Based on the predicted targets for the differentiating miRNA molecules in the psRNA target program, an interactive network showing the miRNA-target connections was constructed using the R’s networkD3 library^40^. For clarity, multiple transcripts of the same gene were collapsed into a single gene node.

To integrate the omics layers, miRNA–target relationships were cross-validated with transcriptome data to identify anticorrelated expression patterns, supported by degradome evidence where available. Differentially expressed genes and metabolites were jointly mapped onto KEGG pathways to highlight overlapping regulatory modules.

## Results

### Results of RNA sequencing

The number of raw sequencing reads and the corresponding number of filtered and trimmed reads for each specific sample is presented in Supplement 2. The trimmed reads were used as the basis for subsequent data analyses, including read alignment, differential expression analysis and pathway enrichment studies.

**Fig. 3:**
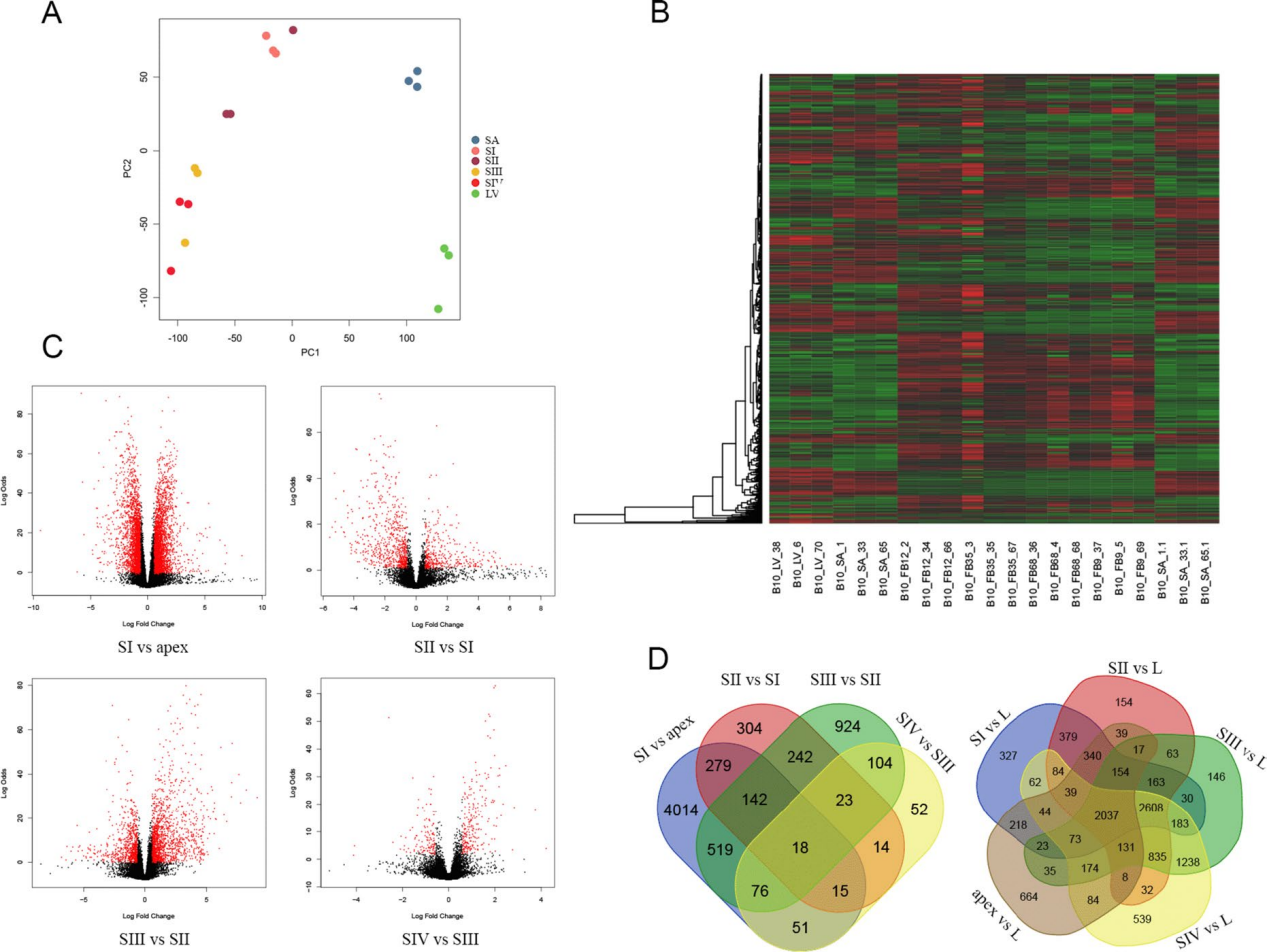
(**A**) Plot showing PCA representation for RNA read counts between leaf (L), shoot apex (SA/apex) and four bud stages: SI, SII, SIII and SIV. (**B**) Heatmap created from a count matrix presenting gene expression in transcripts per million (TPM) between sequenced samples. (**C**) Volcano plots showing the number of differentially expressed (DE) genes for the developmental stages of the buds. (**D**) Venn diagrams showing the number of common DE genes detected between chronological developmental stage comparisons and leaf comparisons.

The mapping of the trimmed reads to the B10v3 reference genome exceeded 90% for each of the samples. The matrix generated by the read counts allows the differences between the sequenced libraries to be visualized using a Principal Component Analysis (PCA) plot (Fig. 3A). The plot of the read counts shows a clear clustering of the samples, notably separating the leaves and the apex with the flower bud clusters by Principal Component 1 (PC1).

### Identification of differentially expressed genes 

The results of the differential gene expression analysis are presented in Table 1 and visualized by volcano plots (Fig. 3C). The highest number of DEGs 5114 were observed in the youngest stage between apex and newly formed flower bud (SI), divided nearly equal among up- and down-regulated genes, 2678 and 2436, respectively. The lowest number of DEGs – 353, is characteristic for SIV vs. SIII comparison, both for up- and down-regulated genes, being 234 and 119, respectively. The heatmap shows the difference in expression profile between sequenced samples (Fig. 3B) while Venn diagrams illustrate the number of genes showing differential expression between the studied groups (Fig. 3D). The results of the differential gene expression analysis are presented in Supplement 3, while the location of DEGs on chromosomes is shown in Supplement 4.

**Table 1:** The number of upregulated and downregulated genes detected in the differential expression analysis for established comparisons based on RNA-seq results.

	Comparison								
	SI vs. Apex	SII vs. SI	SIII vs. SII	SIV vs. SIII	Apex vs. Leaf	SI vs. Leaf	SII vs. Leaf	SIII vs. Leaf	SIV vs. Leaf
Number of upregulated genes	2678	370	1367	234	2190	3596	3899	4423	4477
Number of downregulated genes	2436	667	681	119	1889	3168	3184	3487	3694
Total	5114	1037	2048	353	4079	6764	7083	7910	8171

**Fig. 4:**
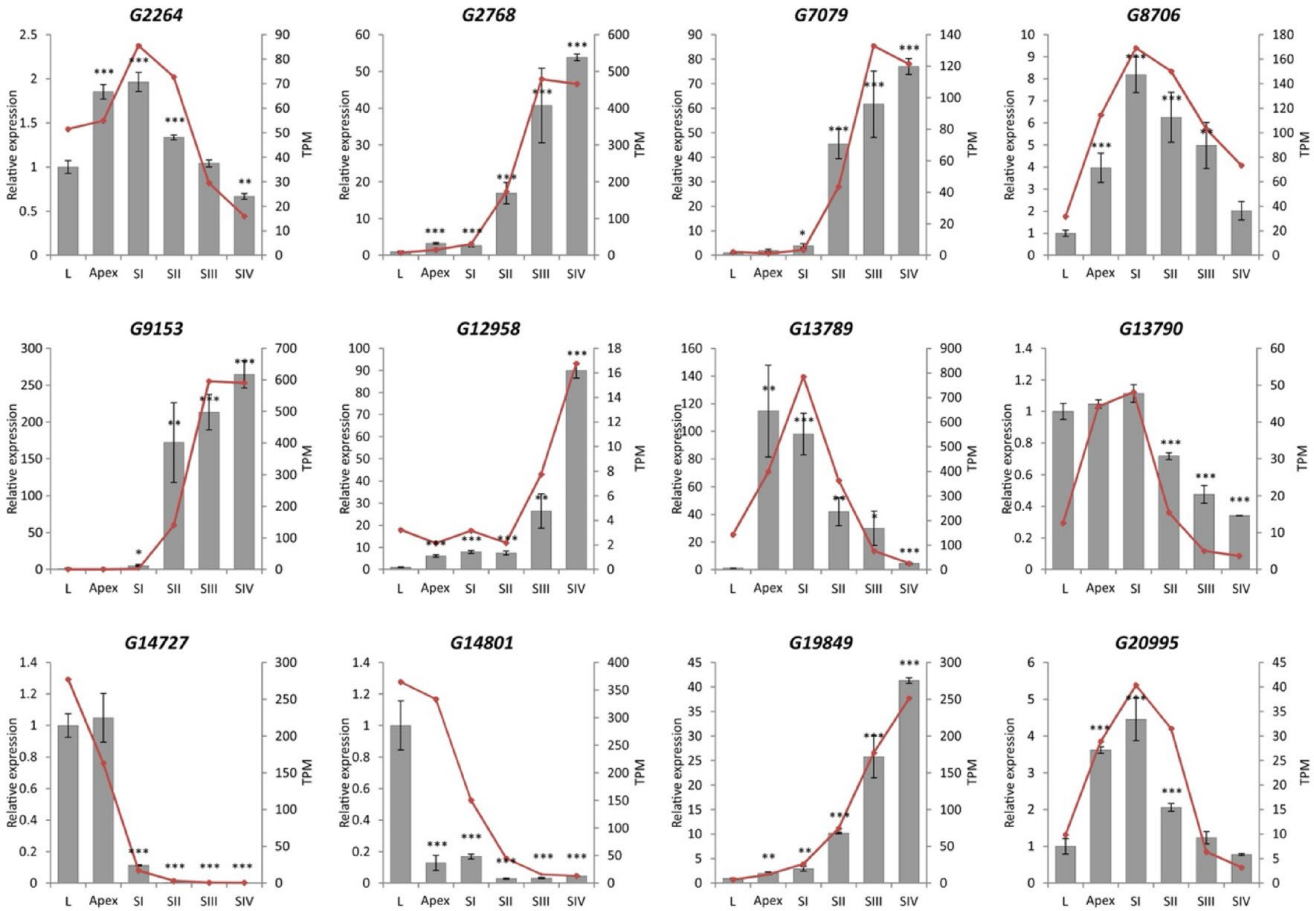
Confirmation of RNA-seq results by qPCR analysis. Gray histograms represent relative expression of genes in apex and flower bud developmental stages compared to leaves (qPCR results) and red points represent bioinformatic results of RNA-seq analysis (in TPM). Significance of the results: *p < 0.05, **p < 0.01, ***p < 0.001.

To confirm the DEGs analysis based on the sequencing data, 12 randomly selected genes were analyzed by qPCR. The transcriptome analysis and qPCR assays showed consistent expression profiles for all genes analyzed (Fig. 4). Low levels of correlation were only observed for a few transcripts, for example, *G13789* or *G13790.*

**Fig. 5:**
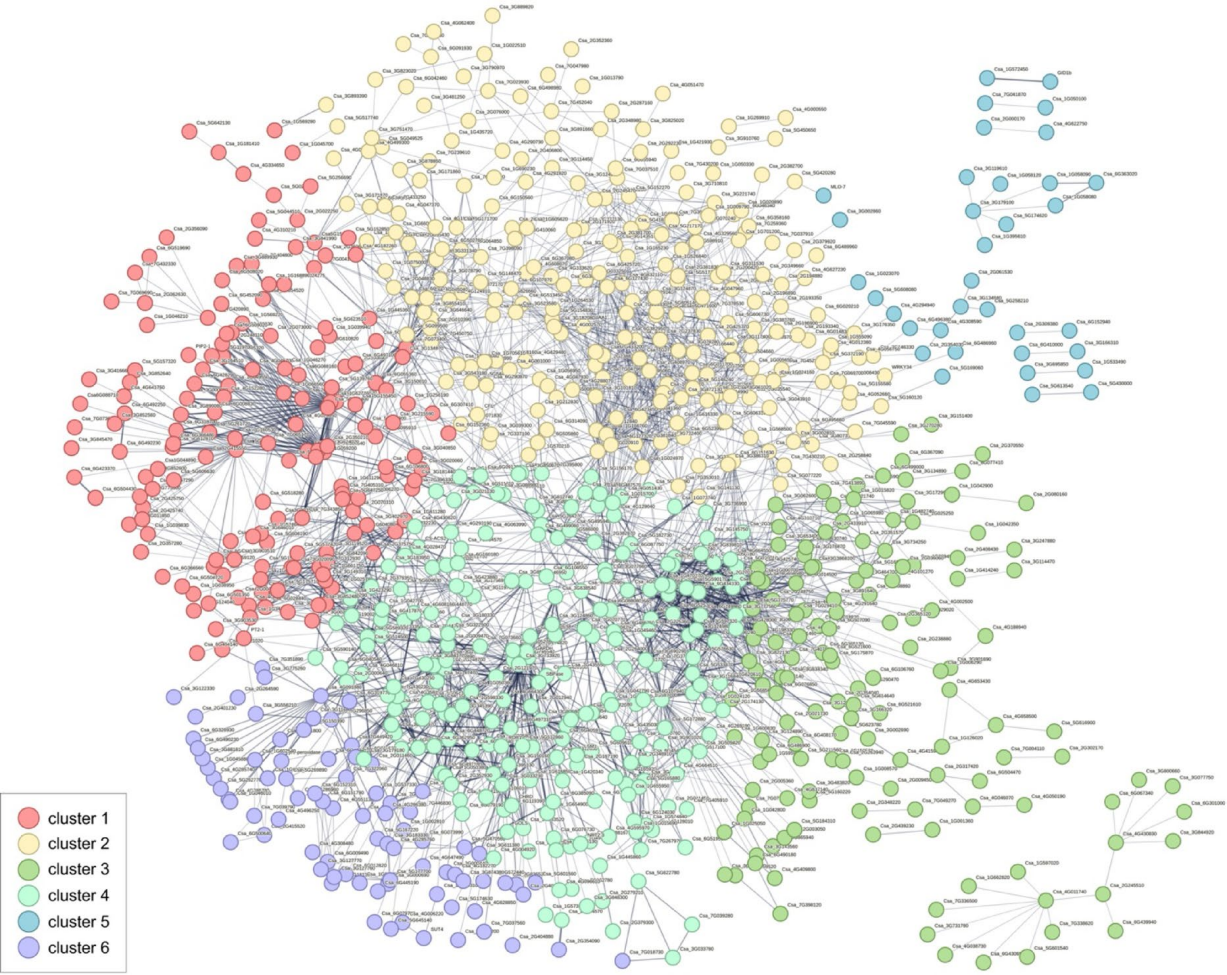
Protein-protein interaction network of DEGs between successive stages of flower development with log_2_FC > 3. Network consists of 1527 proteins connected with 2741 edges, divided into 6 clusters based on the k-means algorithm.

Based on the results of the RNA-seq analysis, genes with log_2_FC > 3 were selected for protein-protein interaction (PPI) network (Fig. 5). The obtained network consists of 1527 proteins, connected by 2741 edges. Proteins were divided into 6 clusters using k-mean clustering. The first cluster consists of 318 proteins mainly involved in catabolic processes, hormone biosynthetic processes and cell wall organization and modification. The second group consists of 286 proteins responsible for transcription regulation, of biosynthetic and metabolic processes. The third cluster consists of 284 nodes, which are engaged in mitotic and meiotic cell cycle processes, chromosome organization and segregation, microtubule and cytoskeleton motor activity. The fourth cluster contains 243 proteins classified to various biological processes with enriched processes like ethylene, amino acids or starch biosynthesis. The fifth cluster consists of 199 nodes involved in binding of clathrin and lipids, while the sixth cluster contains 19 proteins mainly involved in processes associated with lignin and phenylpropanoids.

### GO terms enrichment

**Fig. 6:**
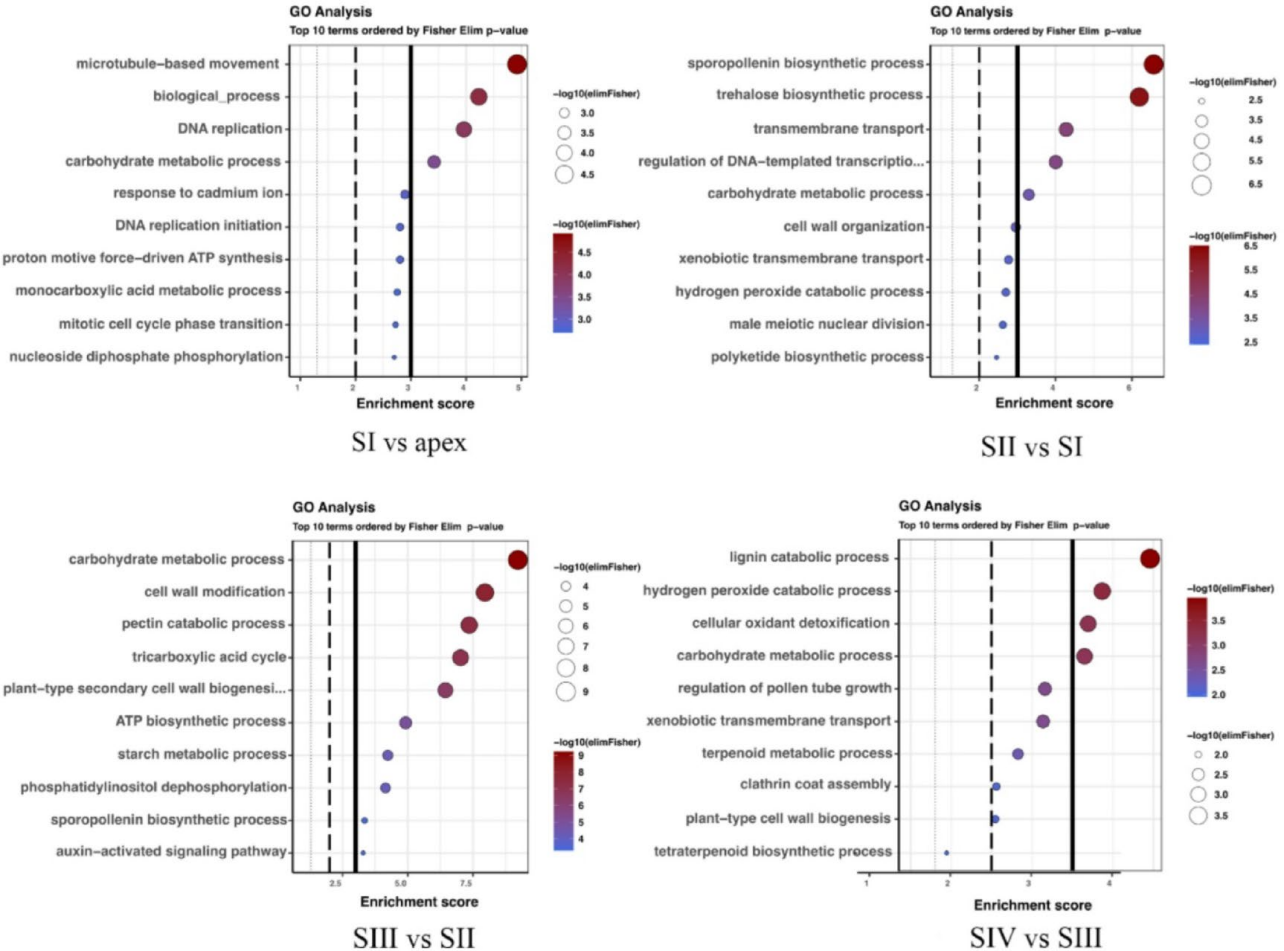
Dot-plot showing enriched ontology terms in bud stage comparisons, SI vs. Apex, SII vs. SI, SIII vs. SII, SIV vs. SIII. Cut-off lines were drawn at the equivalents *p* = 0.05, *p* = 0.01, *p* = 0.001.

The results of the analysis of enrichment in GO terms are presented below in the form of dot plots on Fig. 6 and in Supplement 5. Comprehensive tables with individual enrichment values are provided in Supplement 6. Comparison of the results reveals a single process that was enriched in each of the examined comparisons, namely the carbohydrate metabolic process. The enriched ontological terms detected among genes differentially expressed in overlapping comparisons included starch metabolic process and response to cadmium ion in the SI vs. Apex and SIII vs. SII comparisons, sporopollenin biosynthetic process and transmembrane transport for the SII vs. SI and SIII vs. SII comparisons, hydrogen peroxide catabolic process, cell wall organization, xenobiotic transmembrane transport for the SII vs. SI and SIV vs. SIII comparisons, and lignin catabolic process and plant-type secondary cell wall biogenesis for the SIII vs. SII and SIV vs. SIII comparisons. Other enriched ontological terms detected in comparisons between shoot developmental stages were unique to the examined comparisons. Additionally, processes directly indicative of the characteristics of the male line were enriched, including terms such as pollen exine formation, male meiotic nuclear division, and pollen tube growth. The comparison between generative phases and leaves (Supplement 5) shows enrichment in terms specifically defined as biological processes, suggesting that the differences in such comparisons correspond to highly hierarchical terms in the GO structure.

### Enrichment of KEGG pathways

Results of KEGG^17–19^ pathway enrichment analysis obtained for genes showing differential expression between developmental stages of cucumber flower buds and comparison between flower buds with the leaf are presented in Supplement 7 and Supplement 8. These enriched pathways mainly involved processes related to metabolism, in particular: glyoxylate and dicarboxylate metabolism, carbon metabolism, nucleotide sugar biosynthesis, amine and nucleotide sugar metabolism, starch and sucrose metabolism. The results indicate the involvement of certain pathways in the metabolic dynamics of cucumber bud development, specifically concerning sugar and nucleotide sugar metabolism. Additionally, a consistent pattern of enrichment emerged in the comparisons of different phases of bud growth. In each of these comparisons, a single process, starch and sucrose metabolism, was enriched. This process is responsible for the conversion of complex hydrocarbons into simpler forms. Enrichment of this metabolic pathway in each of the comparisons, as indicated by the GO analysis, suggests that the primary enriched processes in the flower bud development comparisons were those responsible for carbon metabolism. Comparing the enrichment of KEGG pathways between floral bud developmental stages and leaves also suggests the processes of carbon metabolism as the main distinguishing factors in these comparisons.

### Identification of differentially expressed miRNAs 

In the sRNA-seq analysis, potential miRNA molecules were computationally detected for each of the analyzed developmental stages of cucumber flower bud and leaves using the Mirdeep2 and ShortStack programs. The number of raw sequencing reads and the number of reads after the trimming process is presented in Supplement 2.

**Fig. 7:**
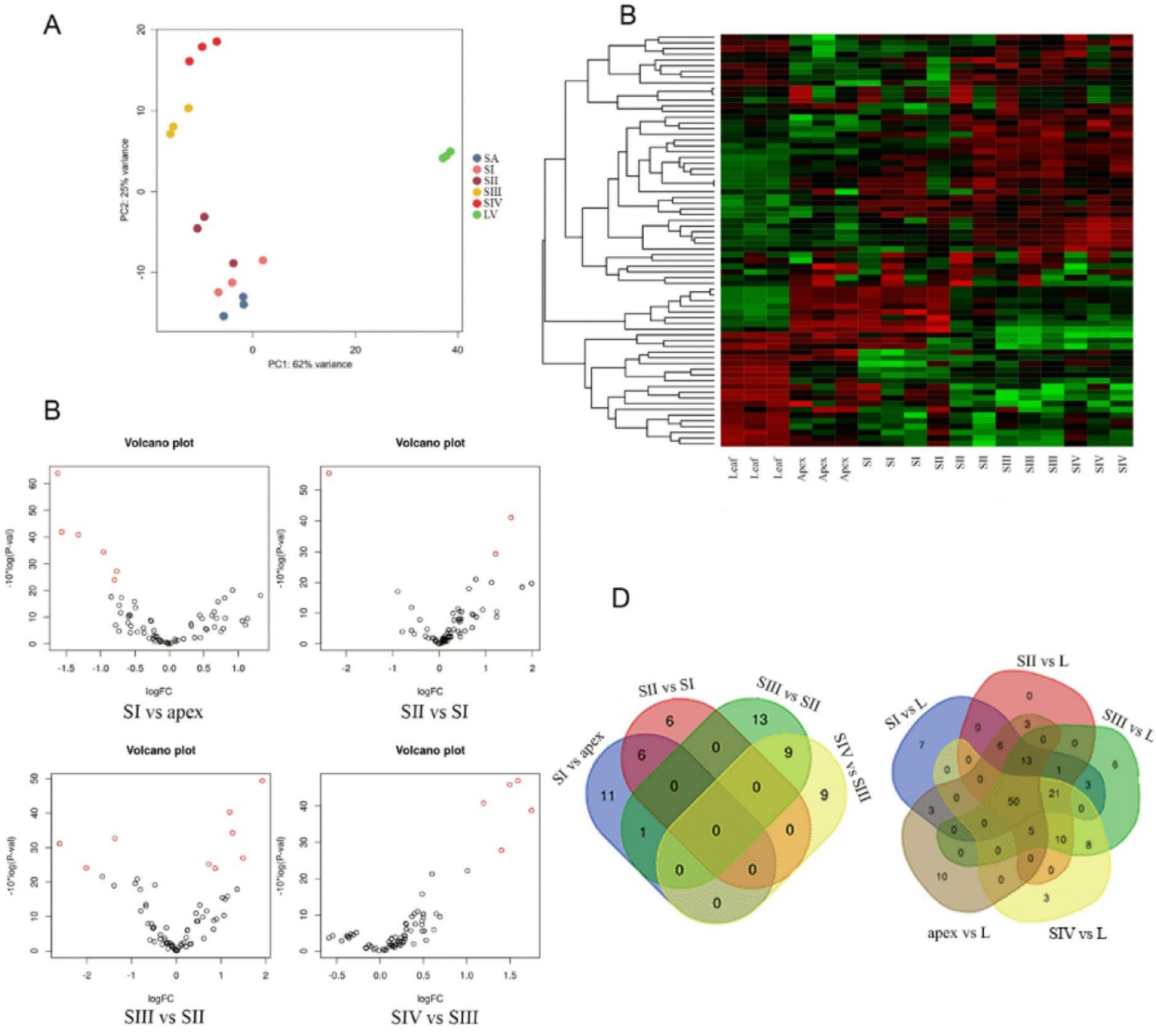
(**A**) PCA plot showing data for the count matrix of all identified sRNA molecules in ShortStack between leaf (L), shoot apex (SA/apex) and four bud stages: SI, SII, SIII and SIV. (**B**) A heatmap showing the miRNA expression profile based on raw miRNA count matrix of the compared samples. (**C**) Volcano plots showing the number of miRNAs undergoing differential expression in comparisons between shoot apex and flower bud growth stages. (**D**) Venn diagrams illustrating the number of shared differentially expressed (DE) miRNAs identified between shoot apex and bud stage comparisons, as well as between bud stage versus leaf comparisons.

Count matrices generated by these two programs facilitated the in silico identification of miRNA molecules with differential expression in selected comparisons, corresponding to the comparisons from the RNA-seq analysis. A representation of the count matrices for the sRNA data is shown in Fig. 7A, presenting a PCA plot of the sRNA molecules for each of the groups under study. It demonstrates that each of the developmental stage groups together and independently. The number of miRNA molecules with a statistically significant differential expression that differed due to the software used, i.e. miRDeep2 and ShortStack, and is presented in Table 2. Notably, given the specific functionality of the ShortStack program and the rigorous evaluation of the detected novel miRNA molecules, novel miRNA discovered by this software was included in the final results.

The results of the differential miRNA expression analysis were also visualized through Volcano plots (Fig. 7C). The heatmap showing the difference in miRNA expression profile between sequenced probes and Venn diagrams illustrating the number of miRNAs that undergo differential expression between the studied groups are presented in Fig. 7B,D, respectively. The results demonstrate that most of the detected miRNAs are developmental stage specific and only a limited number of them are common but related to not more than two stages. The different results are presented for comparisons of developmental stages to leaves, as 49 detected miRNAs are common for all the comparisons.

**Table 2:** The number of differentially expressed miRNA molecules detected. The number represents the count of molecules identified with the same Log_2_FC value. The number within the brackets represents the count of miRNA molecules, encompassing those that differ by a single nucleotide.

	Comparison							
	SI vs. Apex	SII vs. SI	SIII vs. SII	SIV vs. SIII	SI vs. Leaf	SII vs. Leaf	SIII vs. Leaf	SIV vs. Leaf
Number of detected DEmiRNAs with miRDeep2 + edgeR	6 (18)	3 (12)	9 (23)	5 (18)	33 (104)	35 (109)	38 (117)	38 (97)
Number of detected DEmiRNAs with ShortStack + edgeR	3	0	1	4	25	20	38	33
Common	0	0	0	1	10	7	10	7

### Validation of miRNA expression by qPCR

For validation of the bioinformatically identified miRNA molecules, specific criteria were established. miRNA molecules were selected based on their differential expression in several comparisons, and their literature descriptions in other organisms were associated with their involvement in various developmental processes, including sex determination, flower morphogenesis, and fruit development. Additionally, miRNA molecules with identified target transcripts potentially linked to the sex determination process were chosen. Subsequently, isoform sequences for the selected miRNA molecules were compared, thus narrowing the target group of miRNAs for validation. For this refined list of miRNA molecules, primers were successfully designed, and qPCR results were obtained for six different miRNAs. The expression of these miRNAs was assessed at various developmental stages, namely, shoot apex and flower buds of different sizes (SI, SII, SIII, and SIV). Among these miRNAs, miRNA 8 was identified as *novel* in the context of plant biology.

**Fig. 8:**
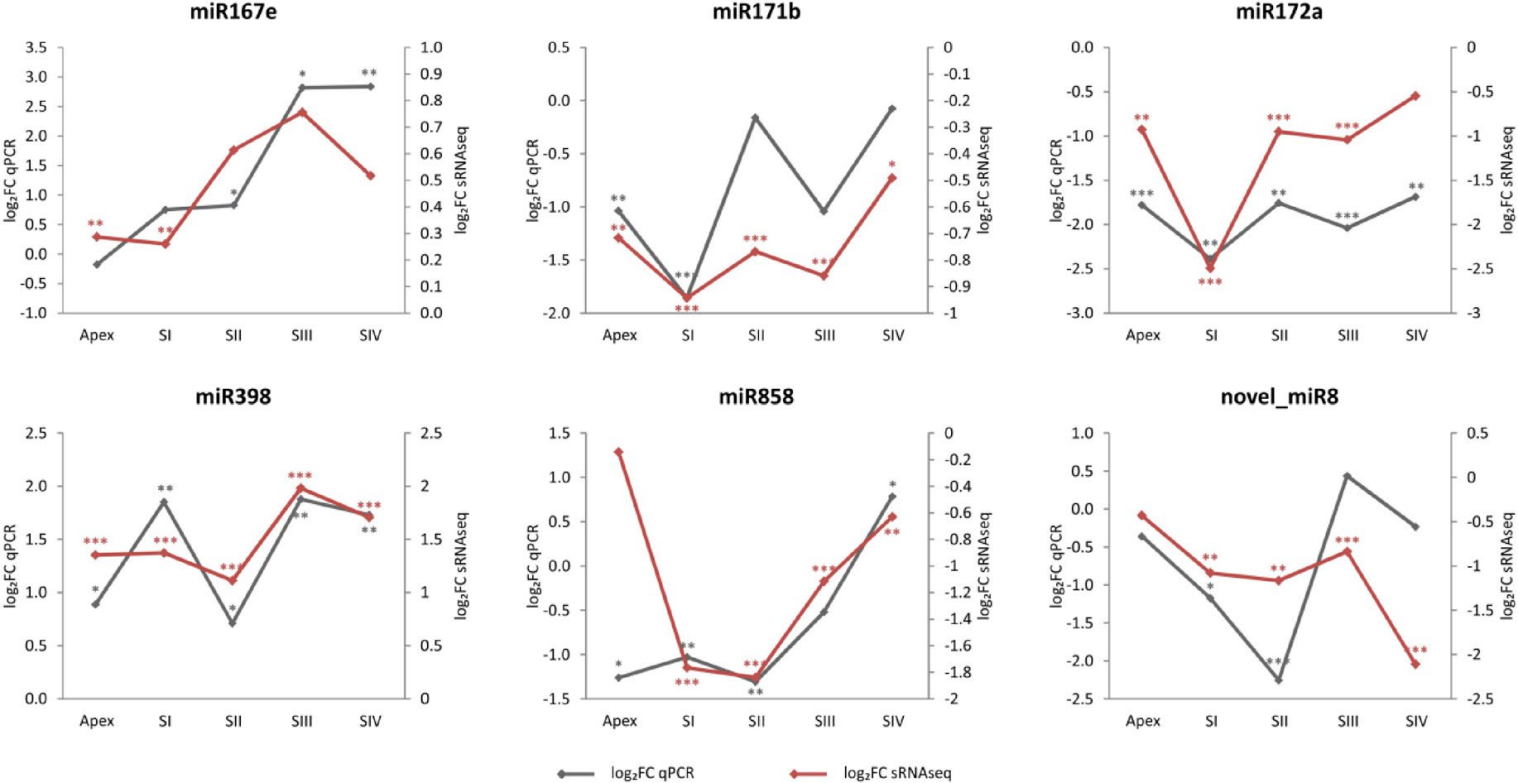
Line plots showing the change in miRNA expression for the floral bud developmental stages versus leaf. The results for qPCR are shown in gray, while the results for bioinformatics analysis are shown in red. Significance of the results: *p < 0.1, **p < 0.05, ***p < 0.01.

The qPCR results were compared with the outcomes of the bioinformatic analysis. The Fold Change values obtained from qPCR were logarithmically transformed and appropriately scaled to set leaf comparison as the baseline. The results of the miRNA differential expression analysis were presented as log₂ fold change (log_2_FC), representing the comparisons between floral bud developmental stages and leaves. Figure 8 illustrates the relative changes in miRNA expression, represented as log₂FC, across individual developmental stages in comparison to leaves. The results of most of the analyzed miRNAs show similar trend in both qPCR and sRNA-seq analysis.

### miRNA’s targets prediction

For the differentiating miRNAs, transcripts of the B10v3 line, which are miRNA targets, were detected with the psRNAtarget server, for which the maximum value of the expectation parameter was 3. A summary of the identified targets for miRNA molecules is provided in Supplements 9 and 10. In the attached spreadsheet, for each of the comparisons examined in the differential miRNA expression assay, columns are included, that describe the transcripts, their annotation, and indicate those transcripts that underwent differential expression in the RNA-seq analysis.

### GO terms enrichment of predicted targets

In the enrichment analysis of target gene sets associated with differentiating miRNA molecules, significantly enriched terms were identified in each comparison. The results are presented in Supplement 11 which depicts the outcomes for the developmental phase of the floral bud, and in Supplement 12, which illustrates the comparisons between floral bud stages and leaves. The detected targets had enriched ontological terms related to the regulation of secondary wall biogenesis and response to salicylic acid, with the comparisons SI vs. Apex and SIII vs. SII showing a high enrichment score for these terms. The term ‘pollen maturation’ had the highest enrichment score in the comparison SII vs. SI, with enriched genes including *Cucsat.G15474* encoding Auxin Signaling F-box 2-like and *Cucsat.G3312* encoding Transport inhibitor response 1. For comparisons SIII vs. SII and SIV vs. SIII, the lignin catabolic process had the highest enrichment score. In comparison SIV vs. SIII, it showed a significant difference compared to the second detected enriched process, floral whorl morphogenesis. The detected enriched processes for floral bud developmental stages indicate differences between the early stages of floral bud development and later ones, where genes leading to the formation of the complete bud structure can be identified. Differences in the detected overrepresented ontological terms correspond to variations in the identified miRNA molecule families for specific bud developmental phases. However, when comparing miRNA targets identified for individual floral bud development stage with those from leaves (Supplement 12), it was observed that there is a substantial similarity between the compared groups in terms of enriched ontological terms. An exception is noted in the comparison between Apex and Leaves, where only two enriched ontological terms were identified.

### Degradome analysis

**Fig. 9:**
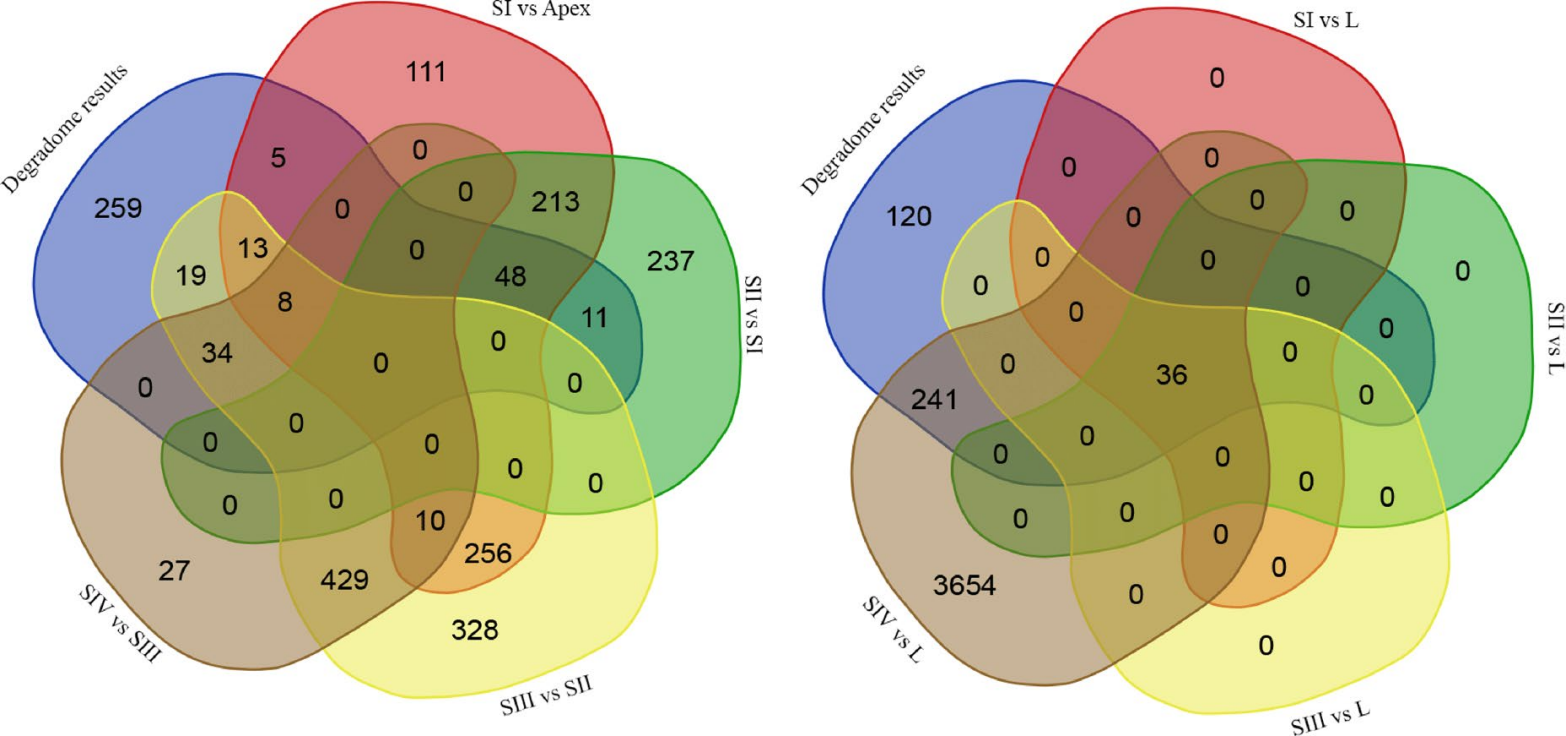
The number of transcripts concurrently identified,  *in silico* using the psRNAtarget program and using degradome sequencing data in the Cleaveland4 program.

The output of the Cleavland4 program resulted in the compilation of a miRNA molecules list, alongside an assessment of potential cleavage sites within the degradome sequencing data (Supplement 13). Consequently, 397 unique transcripts were identified below the statistical significance threshold of the program set at 0.05, corresponding to 85 distinct genes. This list of detected transcripts was cross-referenced with matched targets from the psRNAtarget program. The number of shared transcripts identified both *in silico* and within the degradome sequencing data is presented in Fig. 9.

For comparisons of the detected targets *in silico*, observations parallel those seen in the results of miRNA differential expression analysis. The identified target transcripts exhibit specificity toward distinct developmental phases. However, when compared with leaf, they demonstrate complete overlap. The comparison of results with degradome analysis indicates that, for comparisons across developmental stages of the bud, a certain subset of miRNA targets is confirmed in each analysis. Additionally, it is noteworthy that the number of unique targets identified *in silico* for comparisons SI vs. L, SII vs. L, and SIII vs. L was consistent, totaling 36. Furthermore, all these targets were confirmed in the degradome data. Moreover, *in silico* analysis revealed a significant disparity in the SIV vs. L comparison, where the number of detected unique targets was 3654, while only 36 were validated in the degradome data, akin to the other comparisons. Despite the detection of a substantial number of miRNA transcripts *in silico* in our conducted experiment, degradome analysis revealed a considerable number of transcripts that may be additional subject to regulation by miRNA molecules.

Among the identified transcripts serving as targets for miRNA molecules, it is noteworthy to highlight those transcripts simultaneously identified in multiple comparative analyses (Supplement 14). Such transcripts include, among others, those corresponding to the genes *Cucsat.G12598* (Nuclear transcription factor) - in comparisons SI vs. Apex, SIII vs. SII, SIV vs. SIII; *Cucsat.G5494* (AP2 | Transcription factor); *Cucsat.G15089* (floral homeotic protein APETALA | Transcription factor); *Cucsat.G1761* (floral homeotic protein APETALA) - in comparisons SI vs. Apex, SII vs. SI; *Cucsat.G15511* (Myb transcription factor); *Cucsat.G13338* (Myb-like DNA-binding domain) - in comparisons SI vs. Apex, SIII vs. SII; *Cucsat.G1509* (Nuclear transcription factor Y subunit); *Cucsat.G3007* (Nuclear transcription factor Y subunit) - in comparisons SIII vs. SII, SIV vs. SIII; *Cucsat.G6376* (LITAF-domain-containing protein); *Cucsat.G13151* (Gb protein | 60 S ribosomal protein); *Cucsat.miRNA.G196* (Similar to cme-MIR169l) - in comparisons SI vs. Apex, SIII vs. SII, SIV vs. SIII.

The utilization of degradome sequencing data facilitated the confirmation of results obtained *in silico* through the psRNAtarget program. Consequently, this approach provided validation of the program’s reliability in detecting potential targets for miRNA molecules. Among the potential cleavage sites within transcripts identified in the degradome sequencing data, certain transcripts were recognized, that were detected for specific pairwise comparisons in the analysis of differential miRNA expression. Within this list of transcripts confirmed in the degradome sequencing data, the target transcripts most frequently corresponded to encoded transcription factors, such as AP2 and Myb. These factors were responsible for the most commonly identified targets by the psRNAtarget and Cleaveland4 programs.

### Integrated miRNA–target network analysis

**Fig. 10:**
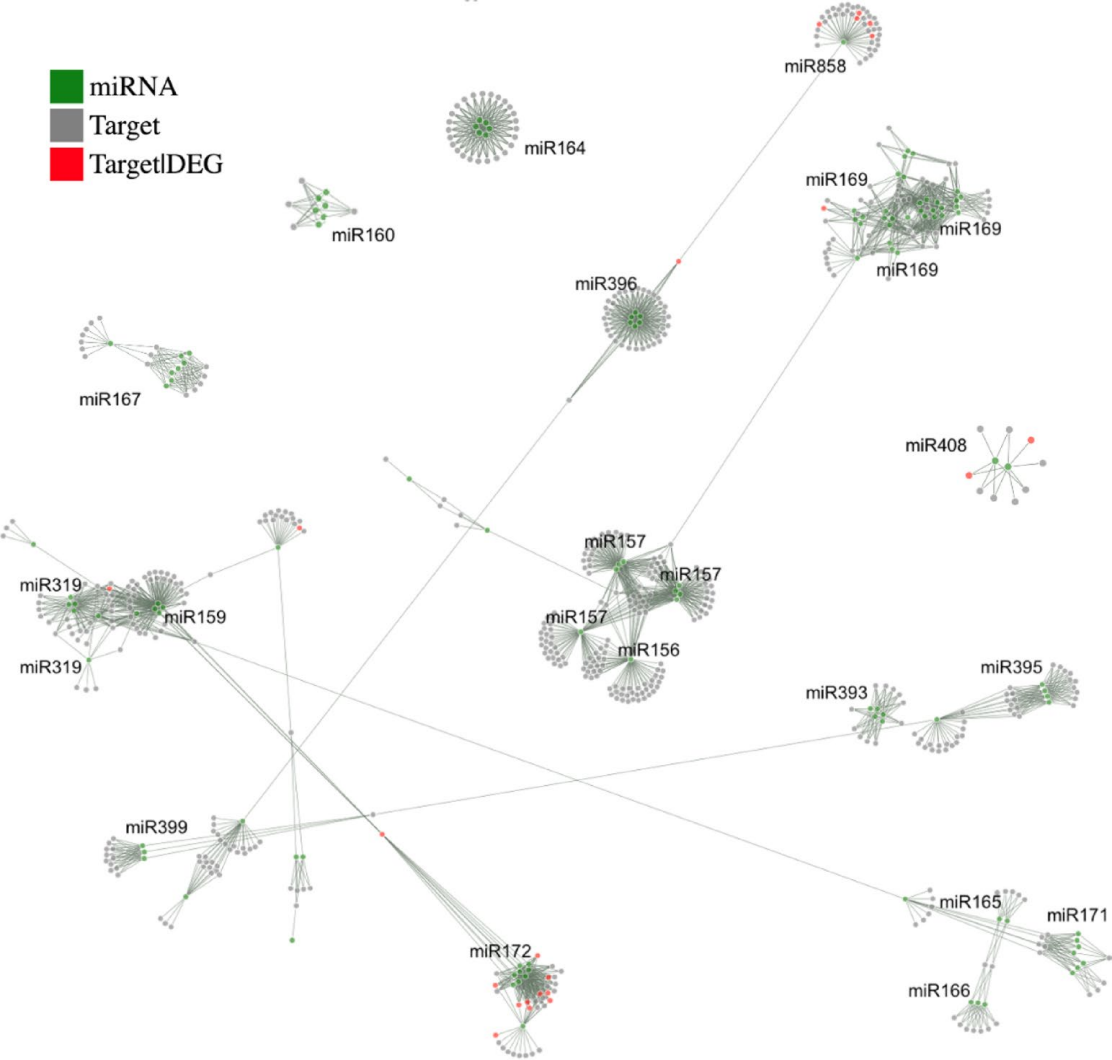
Network of differentially expressed miRNAs and their matched targets. Differentially expressed miRNAs are shown in red; miRNA target transcripts are shown in grey; target transcripts with significant expression changes in RNA analyses are highlighted in red.

To construct a reliable miRNA–target interaction network, candidate targets were first identified using psRNATarget under stringent expectation parameters. To provide experimental confirmation, we integrated degradome sequencing (Cleaveland4), which validated 397 cleavage events and supported numerous predicted interactions, particularly those involving transcription factors and structural genes. The combination of computational prediction and degradome validation thus ensured that the regulatory modules described below reflect genuine biological regulation. Based on this validated dataset, we reconstructed the miRNA–target network and focused on interactions most relevant to male flower development, presented on Fig. 10.

The miRNA-target interaction network has been prepared in HTML format in Supplement 15. It shows miRNA molecules highlighted in green, target genes in grey and target genes that are also differentially expressed (as identified by RNA-seq analysis) in red.

The interactive format of the network allows users to highlight specific miRNAs or genes, search for their names and annotations, reposition individual elements, and adjust the overall network structure. The network contained 147 miRNAs and 632 transcripts, including 23 transcripts with significantly changed expression. A total of 2457 connections were found between miRNAs and transcripts. In analyzing individual elements within the network, it is essential to consider that its construction incorporated all transcripts annotated in the B10v3 cucumber genome, as well as all miRNA molecules showing differential expression. Noteworthy is the presence of several miRNA-target clusters, indicating potential regulation of multiple gene expression by several miRNA molecules. However, a significant portion of the identified potential miRNA-target interactions are specific to individual miRNAs. Nonetheless, between these clusters, individual targets connecting distant clusters can be delineated. This suggests the overall specificity of the action of miRNA families, narrowing potential regulation to genes that will not be regulated by other miRNAs. Individual genes acting as connectors between various miRNAs in the network represent an intriguing aspect in the context of gene expression regulation through miRNA molecules. One of the interesting examples is the gene *Cucsat.G15350*, encoding the transcription factor WRKY, which is targeted by miRNAs from families 396 and 858. Furthermore, *Cucsat.G10816*, which encodes a Myb-like DNA binding domain, acts as a target for miRNA from families 159 and 319 and *Cucsat.G13186* acts as target for miRNA from families 172 and 159. In addition, the map shows a remarkable cluster of genes with altered expression that act as targets for miRNA molecules, particularly in the case of miRNA family 172, where a significant proportion is associated with the APETALA protein.

### Analysis of differentially accumulated metabolites 

As a result of the Metabodiff package execution, DAMs were detected for each of the compared groups. The number of identified differentiating metabolites is presented in Table 3. The numbers in brackets correspond to metabolites that have not been structurally identified and have the category Unknown.

**Table 3:** The number of DAMs detected in the comparisons studied.

	Comparison							
	SI vs. Apex	SII vs. SI	SIII vs. SII	SIV vs. SIII	SI vs. Leaf	SII vs. Leaf	SIII vs. Leaf	SIV vs. Leaf
Number of DAMs	51(29)	19(9)	5(1)	5(3)	114(68)	126(76)	140(86)	127(73)

**Fig. 11:**
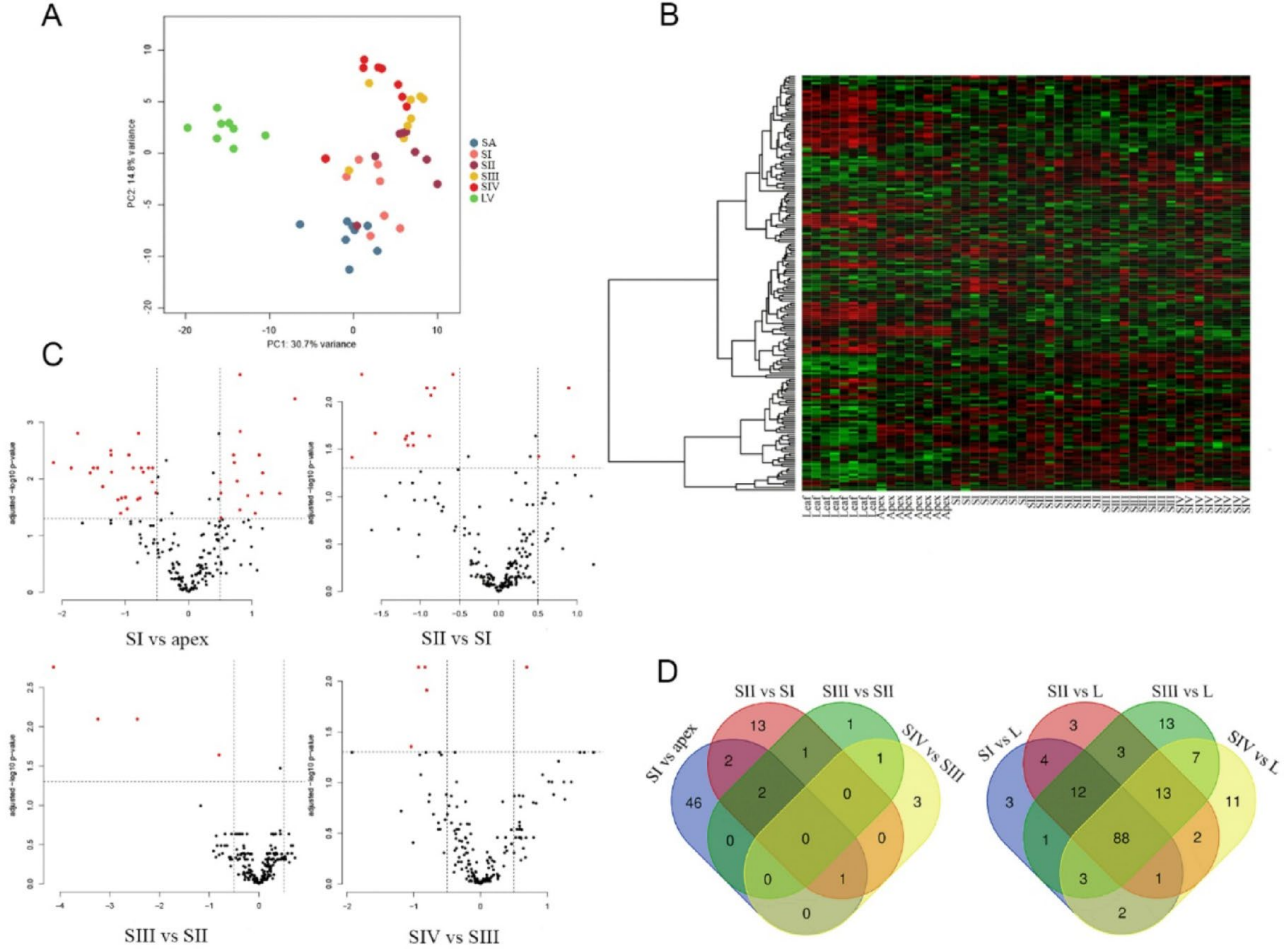
(**A**) PCA plot showing data for a matrix of abundances of the analyzed metabolites between leaf (L), shoot apex (SA/apex) and four bud stages: SI, SII, SIII and SIV. (**B**) Heatmap showing the metabolite level profile of the compared samples. (**C**) Volcano plots of significantly altered metabolites in comparisons between flower bud growth stages. (**D**) Venn diagrams showing numbers of metabolites differently abundant between each comparison.

Metabolites that differentiated more than in one compared group are depicted in the Venn diagrams (Fig. 11D). These diagrams reveal the presence of 88 metabolites collectively distinguishing each floral bud developmental stage from the leaf. Conversely, the comparison between individual phases of bud development indicates greater diversity in DAMs. Hydroxyproline emerged as the principal metabolite detected in the SI vs. Apex, SII vs. SI, and SIII vs. SII comparisons, while proline significantly differentiated in the SII vs. SI and SIII vs. SII comparisons. This underscores the significance of these compounds across successive stages of bud development. Gluconic acid was identified as a significantly DAMs in three comparisons: SI vs. Apex, SII vs. SI, and SIII vs. SII. Other DAMs found in more than one comparison include glycine for SI vs. Apex, SII vs. SI, and SIV vs. SIII, threonic acid and succinic acid for SI vs. Apex and SII vs. SI, and isocitric acid and fumaric acid for SI vs. Apex comparison. Although the number of differentiating compounds between the generative phases and the leaf is substantially higher, there is overlap with the list of metabolites detected in floral bud developmental phases. Comprehensive lists of identified DAMs for the specified comparisons are provided in Supplement 16. Box plots of log_10_ abundance are shown in Supplement 17.

### Integration of results at miRNA-RNA-metabolite level

To address the relationship among the three omics layers, we performed an integrative analysis combining miRNA expression, target gene expression from RNA-seq, and metabolite abundances. This allowed us to identify miRNA–mRNA pairs and to map transcriptomic and metabolomic changes onto common biological processes.

By analysing the results for the expression of detected miRNAs and their targets, we can distinguish 6,741 cases where the expression of the miRNA and its target change in opposite directions (i.e. the miRNA increases when the transcript decreases, or the miRNA decreases when the transcript increases – anti-correlated) from the 5,866 cases where they change in the same direction. Examples of anti-correlation are rare in apical transitions alone, but become more common when compared with the leaf. We identified the following number of such situations: SI vs. L: 1,117 pairs of anticorrelated transcription levels, SII vs. L: 1,335, SIII vs. L: 1,908 (the highest number), SIV vs. L: 1,743, Apex vs. L: 587. In contrast, early apical transitions (e.g. SIII vs. SII) show few or no anticorrelated pairs at the transcript level. This suggests that broad miRNA–mRNA anticorrelation is most clearly evident in leaf comparisons.

Several canonical plant miRNA–target axes dominate the anticorrelated signal. The most frequent of these is the miR169 - NF-Y subunit A axis, which is strongly enriched in leaf comparisons, while the miR396 - GRF (growth-regulating factor) axis also shows prominent enrichment with nearly 300 anticorrelated pairs, particularly in late leaf stages where the miR396a/b families are among the most frequent. The miR160 - ARF (Auxin Response Factors) axis highlights the canonical auxin module, with clear overlaps between the miR160a/b/c families and the ARF transcripts. The miR393 - TIR1/AFB (auxin receptors) axis appears less abundant, but it consistently links auxin receptor genes with the miR393a/b/c families. The miR156/157 - SPL (Squamosa Promoter-Binding-Like) combinations align with the juvenile–adult phase transition module^41^. The miR172 - AP2/ERF axis is detected to a lesser extent; it is active in early apical transitions, but often without corresponding changes at the transcript level. Supplement 18 contains the number of families for which anticorrelated effects of differential microRNA on target transcripts were observed in the RNA-seq results set.

**Fig. 12:**
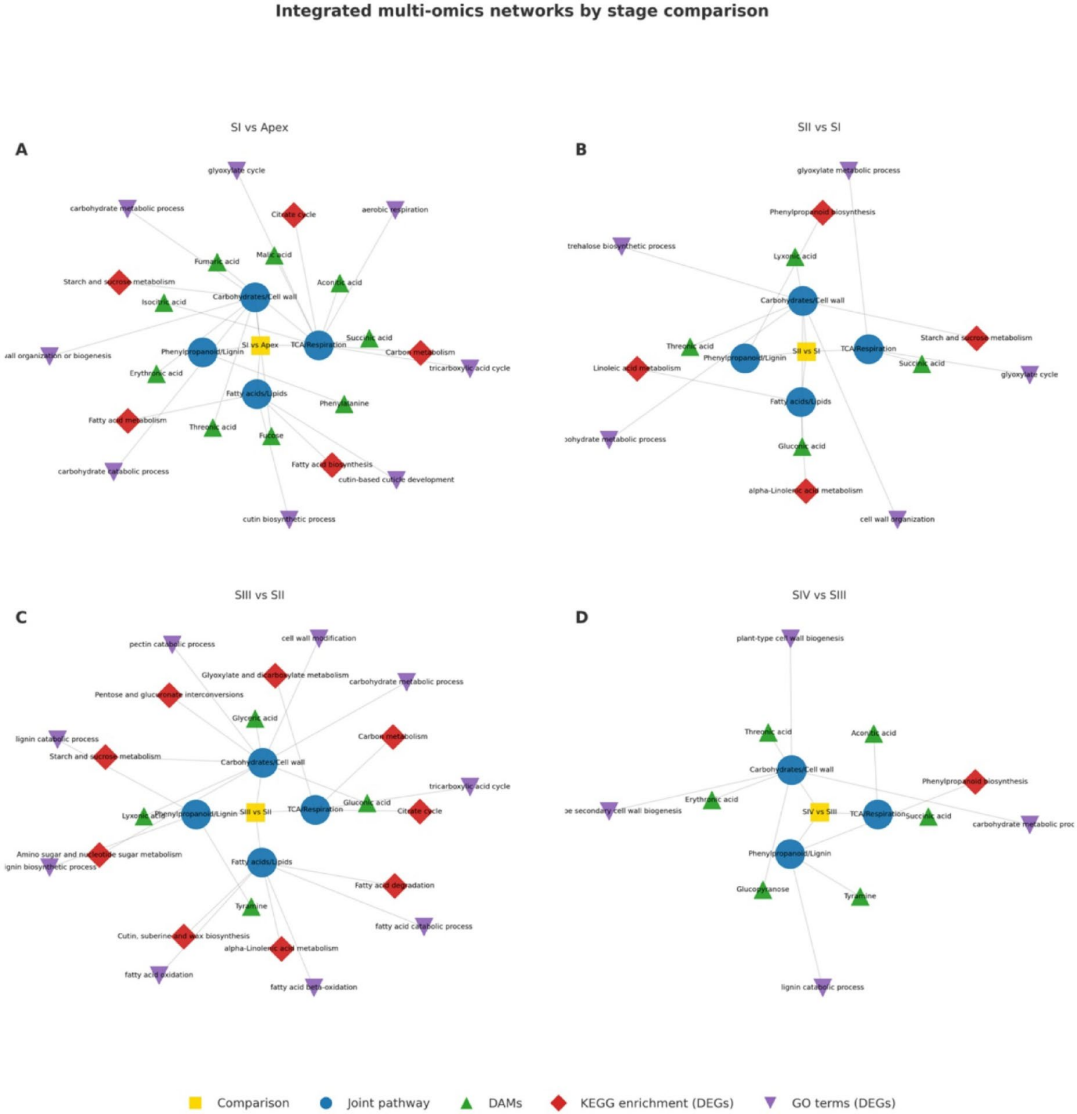
Integrated multi-omics networks of cucumber male flower development. Panels (**A**–**D**) (SI vs. Apex, SII vs. SI, SIII vs. SII, SIV vs. SIII) show stage-specific connections between DEGs, DAMs, and enriched pathways. Nodes represent comparisons (squares), main pathway categories (blue circles), DAMs (green triangles), KEGG terms (red diamonds), and GO terms (purple inverted triangles).

To integrate transcriptomic outcomes with metabolite profiles, correlations were established between functional categories and shifts in metabolic pathways. Genes recurrently represented across enriched GO terms and KEGG pathways were prioritized as central regulators, while metabolite data were mapped onto KEGG pathways to connect ontological functions with observed metabolic changes. This integrative approach enabled functional context from GO annotations to be combined with pathway-level resolution from KEGG, providing a comprehensive framework for linking gene expression dynamics to metabolite variation. We defined jointly altered metabolic pathways as those showing consistent changes in both genes (DEGs) and metabolites (DAMs). (Table 4., Fig. 12).

**Table 4:** Integrated overview of DEGs GO terms and KEGG pathways with DAMs, consolidated as jointly altered pathways. Numbers in parentheses denote the count of differentially expressed genes.

Comparison	Correlation networks comprising DEGs and DAMs			Joint pathway
	DEGs - GO terms (matched)	DEGs - KEGG enrichment pathways (matched)	DAMs	
SI vs. Apex	Tricarboxylic acid cycle (12), aerobic respiration (12), glyoxylate cycle (3)	Carbon metabolism (90), Citrate cycle (TCA cycle) (21)	Succinic.acid, Aconitic.acid.cis., Malic.acid.2.methyl. Fumaric acid, Isocitric acid	TCA/Respiration
SI vs. Apex	carbohydrate metabolic process (241), cell wall organization or biogenesis (102), carbohydrate catabolic process (62)	Starch and sucrose metabolism (55)	Erythronic.acid, Threonic.acid, Fucose	Carbohydrates/Cell wall
SI vs. Apex	No significant enrichment	No significant enrichment	Phenylalanine	Phenylpropanoid/Lignin
SI vs. Apex	cutin biosynthetic process (8), cutin-based cuticle development (6)	Fatty acid metabolism (30), Fatty acid biosynthesis (18)	No differentiation in stage	Fatty acids/Lipids
SII vs. SI	Glyoxylate cycle (2), glyoxylate metabolic process (2)	No significant enrichment	Succinic.acid	TCA/Respiration
SII vs. SI	Trehalose biosynthetic process (7), carbohydrate metabolic process (69), cell wall organization (22)	Starch and sucrose metabolism (21)	Lyxonic.acid, Threonic.acid, Gluconic.acid	Carbohydrates/Cell wall
SII vs. SI	No significant enrichment	Phenylpropanoid biosynthesis (15)	No differentiation in stage	Phenylpropanoid/Lignin
SII vs. SI	No significant enrichment	Linoleic acid metabolism (6), alpha-Linolenic acid metabolism (8)	No differentiation in stage	Fatty acids/Lipids
SIII vs. SII	tricarboxylic acid cycle(13)	Citrate cycle (TCA cycle)(19), Carbon metabolism (48), Glyoxylate and dicarboxylate metabolism (15)	No differentiation in stage	TCA/Respiration
SIII vs. SII	carbohydrate metabolic process (165), cell wall modification (25), pectin catabolic process (52)	Pentose and glucuronate interconversions (38), Starch and sucrose metabolism (36), Amino sugar and nucleotide sugar metabolism (25)	Gluconic.acid, Glyceric.acid, Lyxonic.acid	Carbohydrates/Cell wall
SIII vs. SII	lignin catabolic process (6), lignin biosynthetic process (9)	No significant enrichment	Tyramine	Phenylpropanoid/Lignin
SIII vs. SII	fatty acid oxidation (7), fatty acid beta-oxidation (6), fatty acid catabolic process(6)	Cutin, suberine and wax biosynthesis (10), alpha-Linolenic acid metabolism (13), Fatty acid degradation (11)	No differentiation in stage	Fatty acids/Lipids
SIV vs. SIII	No significant enrichment	No significant enrichment	Succinic.acid, Aconitic.acid.cis.	TCA/Respiration
SIV vs. SIII	carbohydrate metabolic process (28), plant-type cell wall biogenesis (6), plant-type secondary cell wall biogenesis (4)	No significant enrichment	Threonic.acid, Erythronic.acid, Glucopyranose	Carbohydrates/Cell wall
SIV vs SIII	lignin catabolic process (4)	Phenylpropanoid biosynthesis (9)	Tyramine	Phenylpropanoid/Lignin

The enriched gene sets for individual processes include genes that also serve as potential miRNA targets, highlighting the connections between miRNAs, their targets, and associated ontological terms. A total of 156 GO terms exhibiting such correlations were identified; these are listed in Supplement 19, with representative examples shown in Table 5.

**Table 5:** Selected enriched GO processes for differentially expressed genes associated with miRNA–transcript pairs identified in the dataset.

Biological process	miRNA–Target pairs
Anther development	Csa-miR156i – *Cucsat.G13161* Csa-miR156i – *Cucsat.G10617* Csa-miR159a – *Cucsat.G18302* Csa-miR393a – *Cucsat.G3312*
Floral organ development	Csa-miR156i – *Cucsat.G13161* Csa-miR157a – *Cucsat.G13161* Csa-miR159a – *Cucsat.G6174*
Male gamete generation	Csa-miR157a – *Cucsat.G13161* Csa-miR272a – *Cucsat.G17721* Csa-miR156j – *Cucsat.G13161* Csa-miR319a – *Cucsat.G6174*
Pollen development	Csa-miR393a – *Cucsat.G15474* Csa-miR160a – *Cucsat.G17954* Csa-miR393a – *Cucsat.G3312*
Stamen development	Csa-miR157a – *Cucsat.G13161* Csa-miR272a – *Cucsat.G17721* Csa-miR132 – *Cucsat.G14872*
Leaf formation	Csa-miR272a – *Cucsat.G17721*

**Fig. 13:**
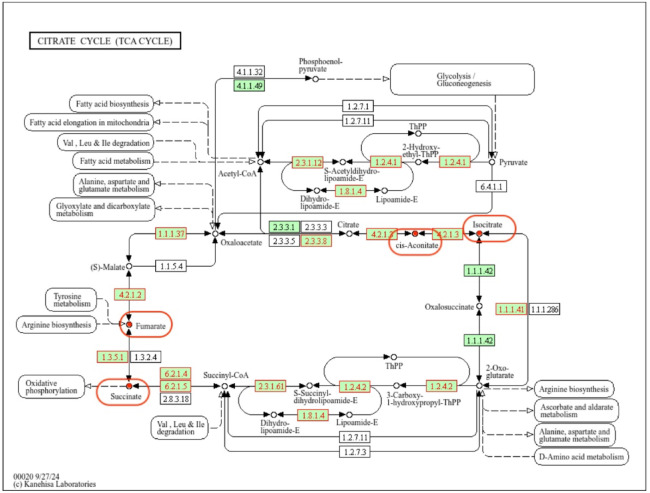
Tricarboxylic acid (TCA) cycle with highlighted DAMs and DEGs identified in the SI vs. Apex comparison.

## Discussion

In this study, we discussed findings at multiple molecular levels — from miRNA–target interactions, through transcriptomic and metabolomic profiles, to their integration — in order to provide a comprehensive view of the regulatory mechanisms underlying male flower development in cucumber.

### Regulatory miRNAs and their targets in flower development

The number of differentially expressed miRNA molecules varied widely in comparisons between the shoot apex and flower buds (SI, SII, SIII, SIV) and between generative organs and leaves. Differentially expressed miRNAs between flower bud stages were unique to specific stages and lacked a common subset, contrasting with the generative organ-to-leaf comparisons where the same miRNAs were consistently differentially expressed. In specific bud development stages, distinct miRNA families were differentially expressed: mir172 and miR169 in SI and SII, with miR393 and miR827 also appearing in SII vs. SI; miR319, miR390, and miR398 in SIII vs. SII; and miR169, miR408, and miR397 in later stages. miRNA-target interactions were validated in cucumber degradome sequencing, confirming miRNA-induced transcript cleavage and supporting bioinformatic predictions.

In order to ensure that the proposed miRNA–target network reflects genuine biological regulation rather than theoretical predictions, we combined computational and experimental approaches. Candidate interactions were first identified using psRNATarget with stringent expectation parameters, and subsequently cross-validated by degradome sequencing (Cleaveland4). The degradome data provided direct evidence of miRNA-guided transcript cleavage, supporting 397 interactions, including several transcription factors and cell wall–associated genes. Together, this two-step strategy forms a robust validation pipeline, allowing us to discuss the regulatory modules identified in our dataset with high confidence. On this basis, we next highlight both conserved regulators (e.g., miR172–AP2, miR169–NF-Y) and novel miRNA (miRNA8) as central components of male flower development in cucumber.

The miRNA molecule from the miR172 family, identified by our study, is a miRNA whose expression has been validated in a two-tailed RT-qPCR experiment, and for which transcripts have been located in the degradome data, specifying the cleavage sites. The miR172, whose target includes the gene *Cucsat.G5494* encoding the transcription factor AP2, undergoes differential expression in the early stages of shoot development. According to the results of bioinformatic analysis, the expression of this miRNA is decreased during the SI growth phase compared to the shoot apex. However, its expression is increased at the SII stage compared to the SI stage and remains at a similar level in the subsequent stages of floral bud development. Comparing the information for miR172 with the literature, in a publication by Heng Lian^42^, the target of miR172 in *A. thaliana* includes the transcription factor AP2. Increased expression of miR172 leads to earlier flowering^42–44^, a phenomenon that has been well described in *A. thaliana*. Furthermore, as described for *A. thaliana*, AP2 inhibits flowering^45^. In our results, a decrease in the expression of the gene *Cucsat.G5494*, encoding the transcription factor AP2, is observed in the developmental stages SI, SII and SIII compared to the leaf and in the comparison of SII with SI. This observation is consistent with the flowering regulation mechanism in *A. thaliana* and suggests a possible occurrence in cucumber. In addition, the gene *Cucsat.5494*, which serves as a target for miR172, was identified in our previous publications as a transcription factor capable of regulating the expression of the cucumber gene *Cucsa.G152620*, which shows differential expression only when comparing male and female cucumber lines^4,44^. Furthermore, this gene is a homolog of the *SKS12* gene in *A. thaliana*, directly associated with the development of the pollen tube and male fertility^46^, indicating the importance of this gene in the process of pollen formation also in cucumber.

In the obtained results microRNA from the miR169 family, exhibits increased expression with the development of the flower buds. Comparing the different phases of the floral bud development, an increase in the expression of this molecule compared to the leaf was observed from the SII phase onwards, except for the SI, where a decrease in the expression of this molecule compared to the leaf and the apex was observed. Among the detected targets of this miRNA molecule, transcripts encoding mainly nuclear transcription factor Y subunit were identified, along with homeobox leucine zipper protein and auxin response factor. It is noteworthy that the nuclear transcription factor Y subunit has been characterized in *A. thaliana* as a transcription factor involved in complexes that initiate photoperiod-dependent flowering^47^. This suggests that in cucumber, nuclear factor Y transcription factors are involved in the process of floral development and their links to changes in miR169 expression.

Our analysis showed also family of miR171, that is involved in different bud developmental stages. Expression of isomers miRNA171b is the highest in SIV bud stage. The miR171 belongs to the conserved family which mainly targets members of SCL which belongs to GRAS family. miR171 assumes a pivotal role in the network governing the regulation of flowering genes through modulation of *SCARECROW-LIKE* (*SCL*) genes (*Cucsat.G114*,* Cucsat.G114*,* Cucsat.G18056*), critical players in meristem development and flower formation. In the context of rice, miR171 precisely targets the cleavage of *SCL* genes, leading to delayed flowering and the consequential generation of sterile spikes, as elucidated by the findings of Curaba et al.^48^. This study sheds light on the molecular mechanisms underlying miR171-mediated control of floral processes and provides valuable insights into the genetic factors influencing reproductive success in rice. In rice the role of miR171 was observed in the phase transition from vegetative to reproductive through OsHAM transcription factors in SAM. Over-expression of miR171 affects phase transitions and floral meristem determinacy in barley^48^.

The upregulation of miR171 expression during the reproductive stage of male flower development showed its potential regulatory mechanisms. These findings presented the contribution to a deeper understanding of the molecular pathways involved in the development of male flowers. The role of miR171-SCL is also observed in floral development through GA signaling and is important for cell meristem proliferation. miR171 target protein LiSCL (*L. longiflorum* Scarecrow-like), also called GRAS, is expressed specifically at the premeiotic phase within anthers^49^.

In our investigation within the plant group, miRNA8 has emerged as novel miRNA. The predicted targets of Csa-novel-miRNA8 included genes encoding enzymes (monocopper oxidase-like protein SKU5 (*Cucsat.G2846*), 6,7-dimethyl-8-ribityllumazine synthase (*Cucsat.G7444)*, 18 S rRNA methyltransferase RID2 (*Cucsat.G15218*)), ribosomal proteins (40 S ribosomal protein S5-like (*Cucsat.G16226*), 50 S ribosomal protein L24 (*Cucsat.G936*)), and regulators of protein modification (RING-type E3 ubiquitin transferase (*Cucsat.G14715*), glycosyltransferase (*Cucsat.G5688*)). Additional targets such as DNA mismatch repair protein MLH3 (*Cucsat.G728*), DEK_C domain-containing protein (*Cucsat.G873*), and kinesin-like protein KIN-12 F (*Cucsat.G891*) suggest involvement in DNA repair, chromatin regulation, and cytoskeletal organization. The above mentioned predicted targets of Csa-novel-miRNA8, suggest a role in sustaining rapid cell proliferation and chromatin organization during early floral bud growth. The discovery of miRNA8 thus adds to the repertoire of small RNAs involved in reproductive development in cucumber and highlights the value of multi-omics approaches for identifying previously unrecognized regulatory molecules.

### Transcriptomic and metabolomic profiles shaping flower development

The enrichment analysis of ontological terms from the differential expression results highlighted connections between specific comparisons during flower bud growth stages. Notably, these terms predominantly indicated the association of gene expression changes with processes related to carbohydrate metabolism (across all generative phase comparisons) and processes involved in starch metabolism. Enriching genes participating in carbohydrate metabolism processes is a pivotal energy source crucial for pollen development^50–52^. Additionally, enrichments in SII compared to SI and SIII compared to SII were linked to sporopollenin biosynthesis, essential for the plant’s outer pollen wall^53^, and male flower development. Furthermore, comparisons between generative phases and leaves revealed enrichments in biological process terms, indicating significant differences corresponding to higher-order terms in the Gene Ontology structure, mainly related to metabolic activities and cellular development. This analysis contributes valuable insights into the molecular mechanisms underpinning the dynamic processes of flower bud growth, particularly emphasizing the intricate interplay of gene expression, metabolic pathways, and cellular development in cucumber plants.

The results of the KEGG pathway enrichment analysis revealed a distinct association of the detected genes with metabolic processes. In comparing the developmental stages of floral buds, three enriched pathways common to groups SI vs. Apex, SII vs. SI, and SIII vs. SII were associated with starch and sucrose metabolism. Additionally, enriched pathways for the tricarboxylic acid cycle (TCA cycle) (Fig. 13) and carbon metabolism were identified in the SI vs. Apex and SIII vs. SII comparisons. These findings align with previous descriptions of metabolic processes during flower development^54^. The KEGG pathway enrichment results, showing enriched pathways, can be aligned with metabolite profiling outcomes, which are discussed in next paragraph. This convergence emphasizes the critical role of carbohydrate metabolism in floral bud development, providing valuable insights into the relevant biological processes and signaling pathways in our dataset.

The significance of carbohydrate metabolism in male tissues becomes evident as sugars play a vital role in the production of fully functional pollen grains, providing the necessary energy for pollen germination and pollen tube growth^55–57^. Maintaining a delicate balance between the continuous supply of carbon sources and the development of reproductive organs is paramount for the formation of flawless flowers. In the context of grapevines, the regulation of carbohydrate metabolism assumes particular importance during normal floral development^58^.

In the process of floral bud development, the regulation of gene expression plays a pivotal role in orchestrating various molecular events. Beyond the well-documented influence of carbohydrate metabolism genes, this study sheds light on the dynamic expression patterns of sporopollenin genes during the initial stages of floral bud formation, specifically in the SI and SII stages . Sporopollenin, a crucial constituent of the inner layer of pollen, is synthesized and stored in the tapetum following meiosis. The present investigation reveals a temporal correlation between heightened expression of sporopollenin genes and the early phases of floral bud development, emphasizing the importance of this process at the critical size range mentioned.

Comparing the numbers of metabolites detected during flower bud growth phases, it can be observed that the highest number of differentiating metabolites was detected in the initial growth phase at the SI vs. Apex, totaling 51 metabolites. In contrast, in later floral bud development stages in comparisons of SIII vs. SII and SIV vs. SIII, this number was only 5. This fact indicates a reduction in the diversity of metabolite levels in successive bud development stages, where initially, a significantly increased change in metabolites is observed. Additionally, the comparison of floral bud developmental phases with leaves in each comparison indicates a number of over 100 differentiating metabolites, clearly distinguishing the metabolites involved in the generative phase of the floral bud from the vegetative phase represented by a mature leaf.

Among the detected metabolites, the significant majority exhibit differentiation across distinct developmental stages of the floral bud. Only a few were identified in more than one developmental stage comparison. Notably, hydroxyproline (trans-4-Hydroxy-L-proline) demonstrated differentiation among three consecutive floral bud development stages, while proline was identified in two comparisons. Hydroxyproline is essential for protein O-glycosylation, a process involving the covalent attachment of glycan chains to the protein backbone, which is critical for glycoproteins that contribute to cell wall integrity^59^. The accumulation patterns of hydroxyproline and proline were consistent with increased expression of HRGP-related genes detected in the transcriptome as DEGs (*Cucsat.G2273*,* Cucsat.G11989*,* Cucsat. G13679*). This agreement between metabolite abundance and gene expression suggests that miRNA–target regulation of cell wall–associated transcripts is functionally linked with metabolite dynamics.

In the context of glycoproteins associated with male gametophyte function, the hydroxyproline-rich glycoprotein (HRGP) family is mentioned, serving various roles in plant growth, development, defense, and signaling^60^, as well as being a component of the calcium signaling pathway^61^. HRGPs are classified into three main families: heavily glycosylated arabinogalactan-proteins (AGPs), moderately glycosylated extensins (EXT), and none or subtly glycosylated proline-rich proteins (PRP)^60,62^. Hydroxyproline is crucial in the context of arabinogalactan proteins (AGPs) and their biosynthesis, which involves O-galactosylation of hydroxyproline (Hyp) residues followed by the stepwise elongation of complex sugar chains^63^. In male gametophytes, AGPs are associated with the control of nexine formation, and their expression is linked to pollen tube growth and pollen viability^64,65^. Hydroxyproline-rich glycoproteins play a role in the plant pollination process, contributing to a diverse array of functions, including the provision of structural integrity and mediation of cell-cell interactions and communication. The process of plant pollination involves a series of interactions between the male gametophyte (pollen grain or pollen tube) and the female gametophyte (pistil) that are thought to signal and regulate the pollen tube growth process, while the extracellular matrix (ECM) of the pistil and pollen tube is enriched in highly glycosylated HRGPs^62^.

In addition, the literature provides information on proline, which also differentiates at early growth stages in the results obtained. Under unstressed conditions, proline accumulates in reproductive organs and tissues, such as flowers, anthers, and pollen grains, suggesting a crucial role for proline in plant reproduction^66^. Furthermore, experiments involving proline-deficient mutants indicated the pivotal role of proline in pollen development and transfer, where the development of gametophytes carrying mutations was severely compromised^67^. In the context of plant sexual reproduction, proline and GABA are particularly associated with pollen vitality and fertility. Specifically, proline can constitute a significant portion, up to 70%, of the free amino acid pool in pollen grains and proline synthesis is essential for proper pollen development and fertility^68^. Furthermore, proline has been linked not only to pollen development, but also to the pollination process itself, where the experiment showed a strong preference of honeybees for proline-enriched nectar, further highlighting the multifaceted role of proline in aspects of sexual reproduction^68^.

The above-mentioned information, published for other organisms^66–68^ indicates the significant role of hydroxyproline and proline in the pollen development process. This, in turn, aligns with the results obtained from our analysis of differential metabolites. The detection of the significance of changes in the levels of hydroxyproline and proline suggests the presence of similarities in the regulation of pollen production between cucumber and *A. thaliana*.

The other detected metabolite, identified in three different developmental stage comparisons, was gluconic acid, which directly contributes to the solubilization of inorganic phosphates, thereby promoting plant growth^69^. Interestingly, gluconic acid, which participates in phosphate solubilization and nucleotide biosynthesis, coincided with differential expression of transcripts (*Cucsat.G9432*,* Cucsat.G14189*,* Cucsat.G13968*,* Cucsat.G16663*,* Cucsat.G3792*) involved in carbohydrate metabolism and energy supply, including KEGG pathways such as the TCA cycle and starch/sucrose metabolism. Noteworthy is the glucose-to-ribose conversion pathway, in which gluconic acid serves as an intermediate product^70^. Ribose, in turn, is directly associated with developmental processes through its involvement in nucleotide biosynthesis^71^.

A detailed analysis of the developmental stages of the flower bud revealed a distinct group of differential metabolites, with the highest number (51) identified in the comparison between SI vs. Apex. This finding suggests that early flower bud development is associated with the increased regulation of metabolic activity. Among the 46 unique differential metabolites detected in this comparison, phenylalanine influences flower bud development^72^. Similarly, methionine plays a pivotal role in ethylene biosynthesis^72^, while fumaric acid and aconitic acid serve as key intermediates in the TCA cycle^73,74^. Regarding gene expression, comparing SI to Apex revealed a significantly higher number of gene changes (4014) compared to other developmental stages. The enrichment analysis for this comparison of KEGG pathways revealed a significant activation in processes such as carbon metabolism, fatty acid metabolism, DNA replication and the citrate cycle. Additionally, Gene Ontology analysis underscored the importance of carbohydrate metabolic processes, monocarboxylic acid metabolism, and DNA replication during this phase. Collectively, these findings underscore the Apex-to-SI growth phase as a period of intense metabolic and transcriptional activity. Several genes exhibiting significant expression changes in this stage were also confirmed using the qPCR method and matched RNA-seq results. These include *Cucsat.G2768*, *Cucsat.G13789*, *Cucsat.G14727*, *Cucsat.G14801*, and *Cucsat.19,849*.

To further characterize the male cucumber line, we can refer to the results obtained in the publication by Zeyu Cai et al.^70^, where the authors propose that male flowers exhibit increased carbohydrate consumption compared to female flowers. Indeed, among the detected differentiating metabolites, we can highlight those directly involved in the Tricarboxylic Acid (TCA) cycle or serving as precursors to compounds participating in this process. Such metabolites include fumaric acid and isocitric acid, identified as differentiating compounds in the SI vs. Apex comparison, as well as succinic acid, detected in the SI vs. Apex and SII vs. SI comparisons. It is noticeable that these differentiating metabolites are primarily identified at the initial stages of floral bud development. Sugar metabolism has been indicated to play a crucial role in male reproduction and is directly associated with pollen development and male fertility^51^.

In addition, reproductive development has been identified as more susceptible to abiotic stress compared to vegetative stages, particularly during the phases of seed and fruit formation^75^. Sucrose metabolism plays a crucial role in this context, being described as pivotal for development, stress response, and yield formation. Additionally, sucrose metabolism is implicated in signaling pathways by generating various sugars as metabolites, driving growth, and synthesizing essential compounds^75^. In a study focusing on pollen tubes in *Nicotiana tabacum*, sucrose was highlighted as a compound providing the necessary energy for pollen tube elongation and development. Furthermore, the presence of sucrose was deemed indispensable for optimal pollen germination and pollen tube length during specific growth periods^76^.

In summary, transcriptome and metabolite profiling suggest a key role for carbohydrate metabolism, amino acid dynamics, and pollen wall biosynthesis in male flower development. The coherence between gene expression and metabolite changes, such as the parallel regulation of HRGP-related genes with hydroxyproline/proline accumulation, highlights the functional connections between transcriptional and metabolic levels. These observations provide a solid foundation for integrative analyses in which the interplay of miRNAs, transcriptome programs, and metabolite dynamics can be assessed to create a comprehensive regulatory framework for cucumber male flower morphogenesis.

### Integration of results across miRNA, transcriptome, and metabolome

While individual omics layers provided important insights into regulatory mechanisms of male flower development, their integration reveals how these processes are coordinated across molecular levels. By linking miRNA–target interactions with transcriptomic shifts and corresponding metabolite changes, we were able to uncover regulatory cascades that control developmental transitions more comprehensively than single-omics analyses alone. The following section presents these integrative findings, highlighting conserved and novel modules that jointly orchestrate cucumber male flower morphogenesis.

Integration of the three omics layers revealed coordinated regulation of floral bud development. miR172 showed stage-specific shifts (SI–SII) targeting AP2 (*Cucsat.G5494*), with transcriptomic data confirming reduced AP2 expression across SI–SIII, consistent with the conserved miR172–AP2 module in *A. thaliana*. Similarly, rising miR169 levels correlated with downregulation of NF-Y subunits, while miR171 aligned with suppression of SCL/GRAS transcription factors, supporting known roles in meristem regulation. These anticorrelated miRNA–target pairs strengthen confidence in direct regulatory interactions and highlight key developmental switches.

At the metabolic level, hydroxyproline and proline correlated with expression of HRGP genes *(Cucsat.G2273*,* Cucsat.G11989*,* Cucsat.G13679*), reinforcing their role in pollen viability, fertility, and cell wall integrity. Carbohydrate metabolism emerged as another convergence point: transcriptomic enrichment highlighted starch and sucrose metabolism across SI–SIII, while metabolite profiling detected TCA intermediates (fumarate, aconitate, succinate) essential for energy supply during pollen development. This overlap underscores carbohydrate metabolism as a central driver of floral growth.

Stage-specific integration identified SI vs. Apex as the most dynamic transition, with the largest number of differentially expressed genes (4014), miRNA changes (including miR172), and metabolites (51). Later stages (SIII vs. SII, SIV vs. SIII) exhibited fewer differences, indicating stabilization of developmental programs as reproductive structures matured.

Together, these findings outline a coherent model: miRNAs function as upstream regulators of transcription factors, transcriptomic changes execute developmental programs, and metabolites capture the biochemical shifts sustaining growth, energy demand, and fertility.

These results illustrate a consistent regulatory cascade: (i) miRNAs modulate the expression of transcription factors (e.g., AP2, NF-Y, GRAS/SCL), (ii) transcriptomic changes activate pathways related to carbohydrate metabolism, sporopollenin biosynthesis, and cell wall organization, and (iii) these pathways are reflected at the metabolite level by shifts in hydroxyproline, proline, and TCA intermediates. Such cross-validation across omics levels reinforces the robustness of our findings and provides a comprehensive framework for understanding male flower morphogenesis in cucumber.

Distinct molecular signatures characterized successive transitions: Apex to SI showed intense cell division, respiration, carbohydrate turnover, and ROS detoxification; SI to SII reflected storage carbohydrate metabolism, lipid signaling, and initiation of meiotic and pollen wall programs; SII to SIII featured peak polysaccharide metabolism, secondary wall remodeling, high respiration, ascorbate-linked redox activity, and pollen tube signaling; while the SIII to SIV transition carried a maturation signature with activation of carotenoid, ABA, and phenylpropanoid pathways, continued wall assembly, and increased transport and phosphorylation activity.

This integrative approach not only validated known regulatory modules (e.g., miR172–AP2, miR169–NF-Y) but also revealed metabolic convergence points such as hydroxyproline/proline accumulation aligned with HRGP gene expression. Such multi-omics integration highlights central processes that would remain hidden in single-omics analyses.

The conducted analyses have yielded results at various omics levels, the compilation and interpretation of which pose a particular challenge. Flower bud development is a complex process orchestrated by intricate interactions among the transcriptome, miRNome, and metabolome. Transcriptomic analyses have identified numerous genes and transcription factors that regulate floral initiation and progression. Overlap analyses offered insights into the global regulatory impact of miRNA on gene expression, demonstrating miRNA-mediated modulation of target genes. Additionally, an interactive network of miRNA-target interactions was developed, enabling the identification of miRNA clusters and multi-target regulation, thereby simplifying data presentation and offering new perspectives. Metabolomic studies complement these findings by elucidating the biochemical pathways active during bud development. Integrating transcriptomic, miRNome, and metabolomic data provides a holistic understanding of the molecular mechanisms governing flower bud development in cucumber B10 line. These comprehensive analyses offer valuable insights into the genetic and biochemical networks that control floral development, facilitating targeted breeding strategies and genetic improvements that could be appliedd in various plant species.

To sum up, integrating miRNA, transcriptome, and metabolome datasets revealed a multilayered regulatory framework controlling male flower development in cucumber. Conserved modules such as miR172–AP2 and miR169–NF-Y act alongside novel miRNAs to fine-tune transcriptional programs, while metabolic shifts in hydroxyproline, proline, and carbohydrate pathways provide the energy and structural support required for pollen formation. This cross-validation across omics levels not only confirms known mechanisms but also uncovers previously unrecognized regulatory cascades, offering a comprehensive model of male flower morphogenesis and a foundation for future functional and breeding studies.

## Conclusion

The analyses of high-throughput sequencing data presented in this study provide new insights into the molecular basis of male cucumber flower development. By combining transcriptomic, small RNA, and metabolomic approaches, we expanded the knowledge of cucumber flower morphogenesis at multiple regulatory levels. Analysis of miRNA families identified conserved regulators such as miR172, miR171, and miR169, while novel miRNA candidate add further layers of regulation. Integrating miRNA–target networks with transcriptomic and metabolite data highlighted carbohydrate metabolism, sporopollenin biosynthesis, and amino acid dynamics as central processes supporting pollen formation and fertility. Together, these findings establish a comprehensive framework linking small RNAs, transcriptional and metabolites profiles, offering new perspectives on cucumber sex determination and providing a foundation for future functional studies and breeding applications.

## Supplementary Information

Below is the link to the electronic supplementary material.

Supplementary Material 1

## Data Availability

All clean reads generated by Illumina sequencing have been deposited in the Sequence Read Archive database ( [http://www.ncbi.nlm.nih.gov/sra] (http://www.ncbi.nlm.nih.gov/sra) under BioProject PRJNA1166086.
