# Supplementary material for: Multi-omics integration of transcriptome, miRNA, and metabolome uncovers molecular mechanisms of male flower development in cucumber line B10 (Cucumis sativus L.)
Source: Sci Rep. 2025 Nov 29;15:45734. doi: 10.1038/s41598-025-28485-6 (PMC12753748; doi:10.1038/s41598-025-28485-6)

Selected target plot (T-plot) charts, generated using the CleaveLand4 program, depict the identified cleavage positions of selected transcripts by miRNA molecules. The position flagged with the red dot was found with highest frequency.


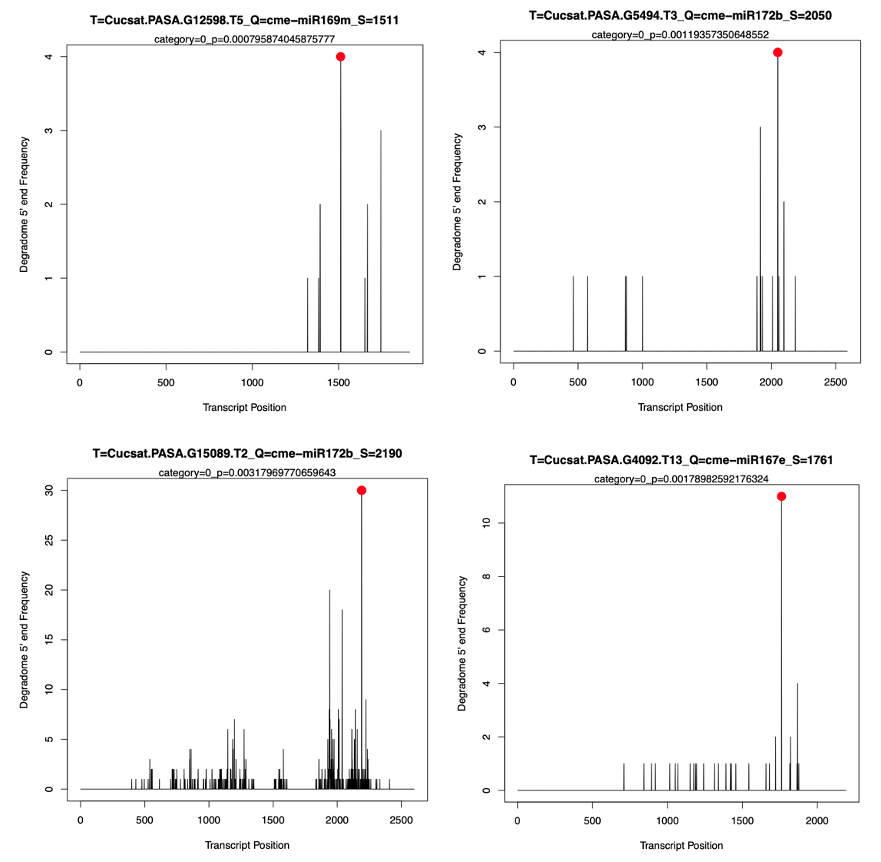

Supplement: Supplementary file 1 — Supplementary Material 1 [file 41598_2025_28485_MOESM1_ESM.zip › Supplements_B10/S14.Selected_Target_plots.docx]
