# Supplementary material for: Multi-omics integration of transcriptome, miRNA, and metabolome uncovers molecular mechanisms of male flower development in cucumber line B10 (Cucumis sativus L.)
Source: Sci Rep. 2025 Nov 29;15:45734. doi: 10.1038/s41598-025-28485-6 (PMC12753748; doi:10.1038/s41598-025-28485-6)

A graph presenting the most abundant miRNA families for which anti-correlated miRNA-target pairs were found.


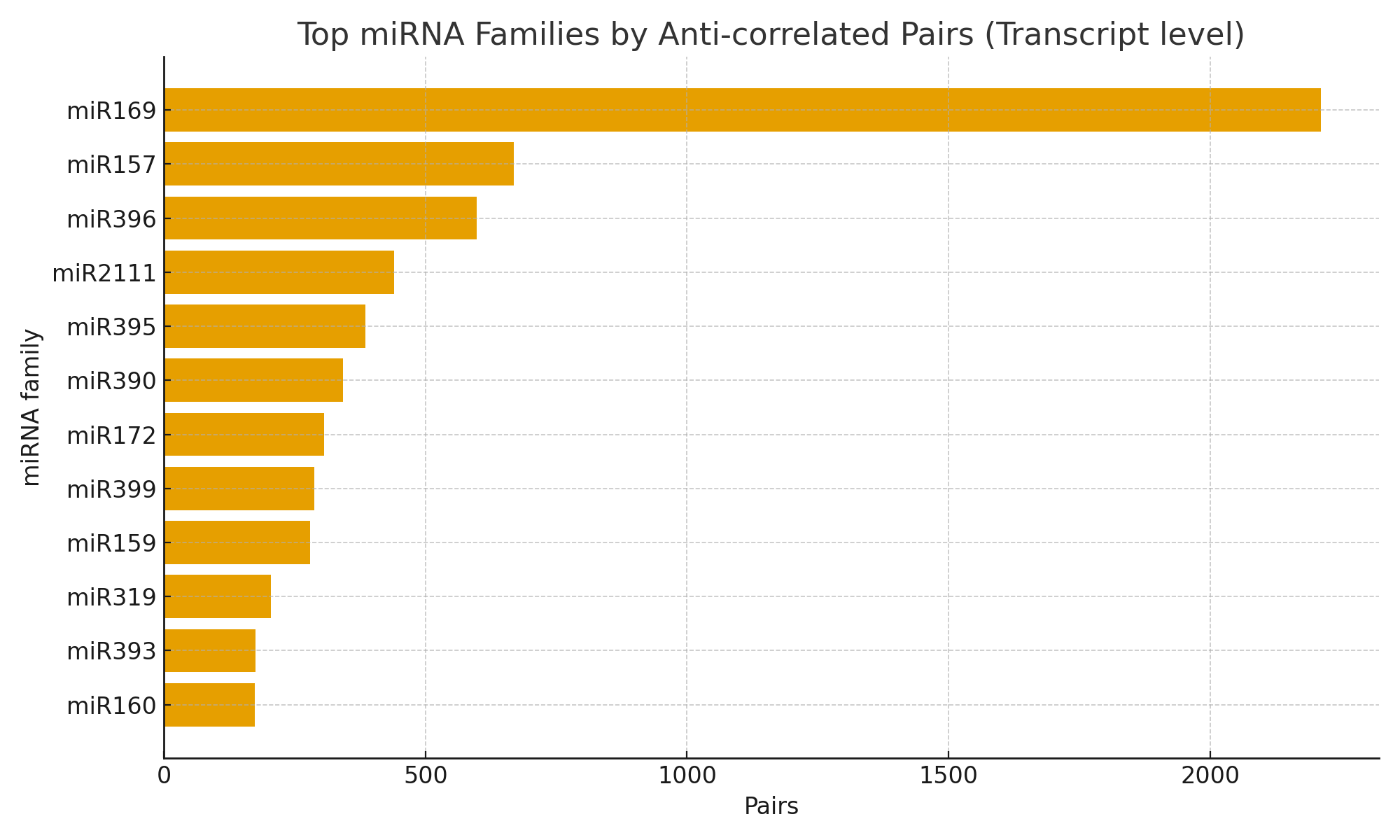

Supplement: Supplementary file 1 — Supplementary Material 1 [file 41598_2025_28485_MOESM1_ESM.zip › Supplements_B10/S18.miRNA_families.docx]
