# Supplementary figures and images for: Multi-omics integration of transcriptome, miRNA, and metabolome uncovers molecular mechanisms of male flower development in cucumber line B10 (Cucumis sativus L.)
Source: Sci Rep. 2025 Nov 29;15:45734. doi: 10.1038/s41598-025-28485-6 (PMC12753748; doi:10.1038/s41598-025-28485-6)

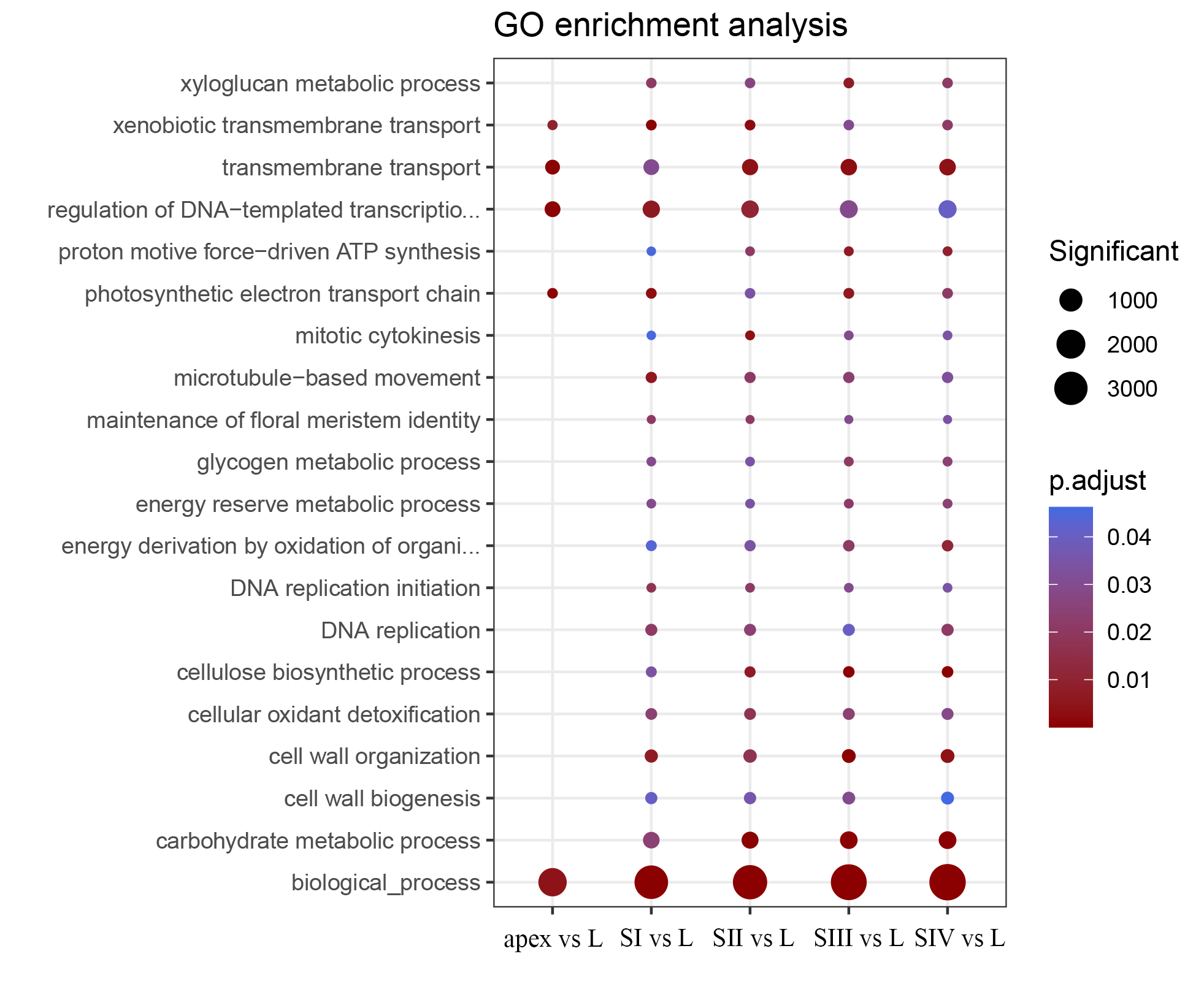

Supplement: Supplementary file 1 — Supplementary Material 1 [file 41598_2025_28485_MOESM1_ESM.zip › Supplements_B10/S5.GO_enrichment_leaves.png]

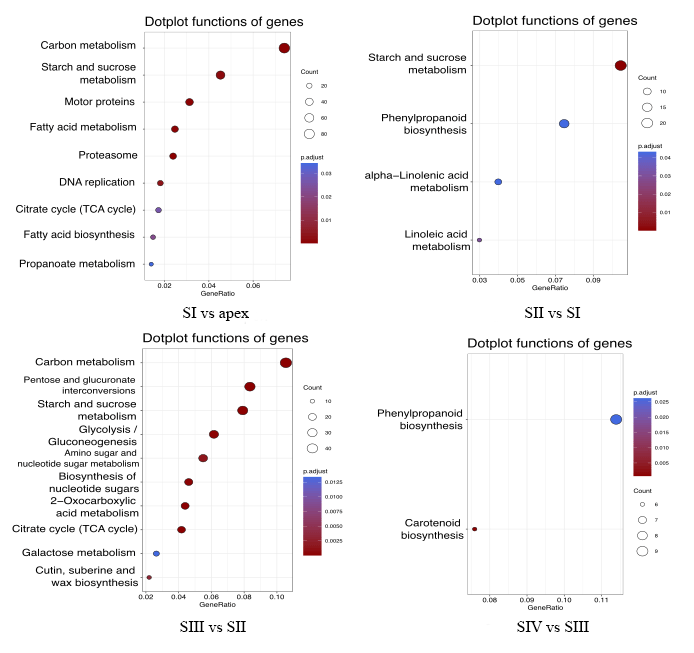

Supplement: Supplementary file 1 — Supplementary Material 1 [file 41598_2025_28485_MOESM1_ESM.zip › Supplements_B10/S7.KEGG_enrichment_dev_stages.png]

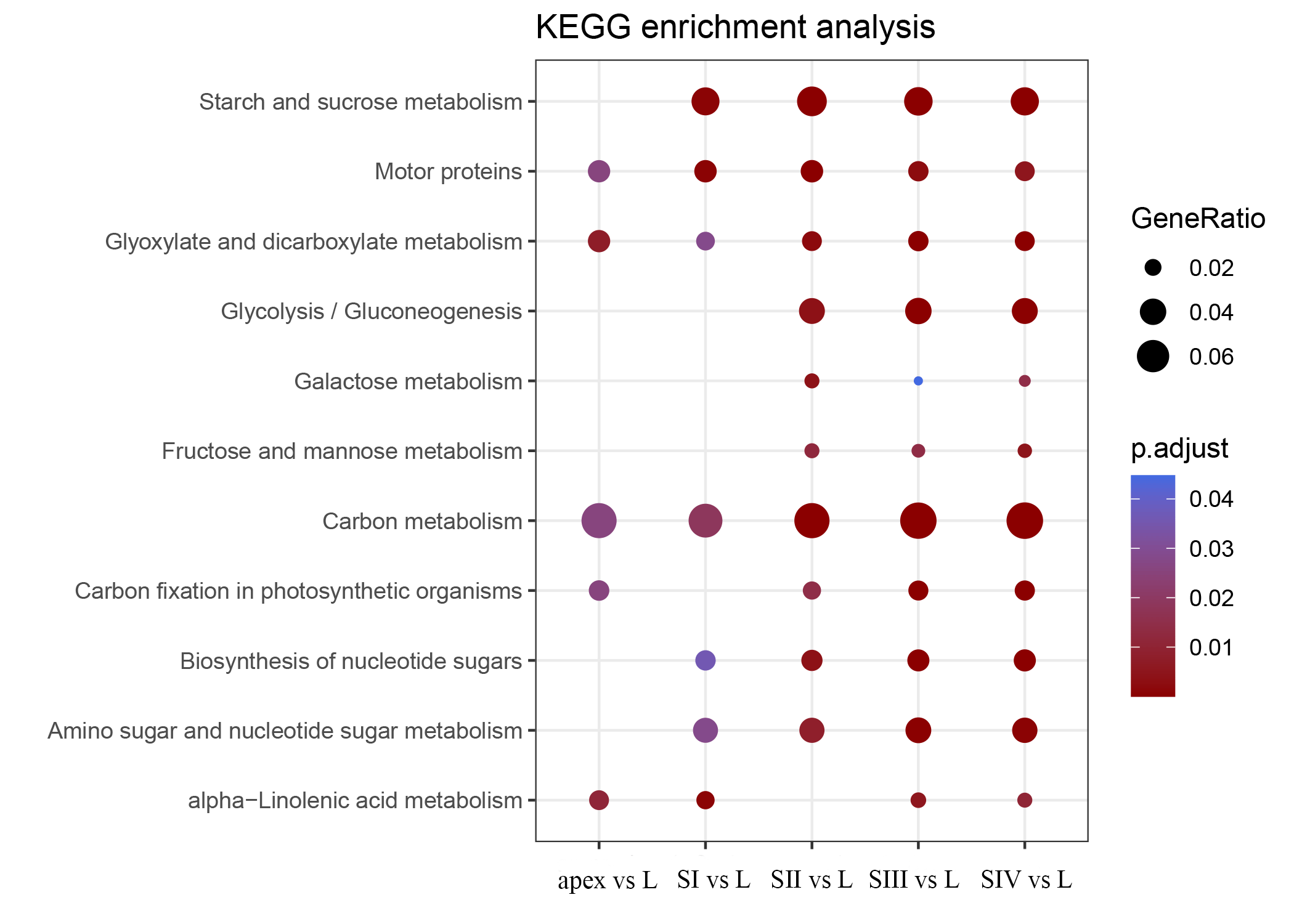

Supplement: Supplementary file 1 — Supplementary Material 1 [file 41598_2025_28485_MOESM1_ESM.zip › Supplements_B10/S8.KEGG_enrichment_leaves.png]

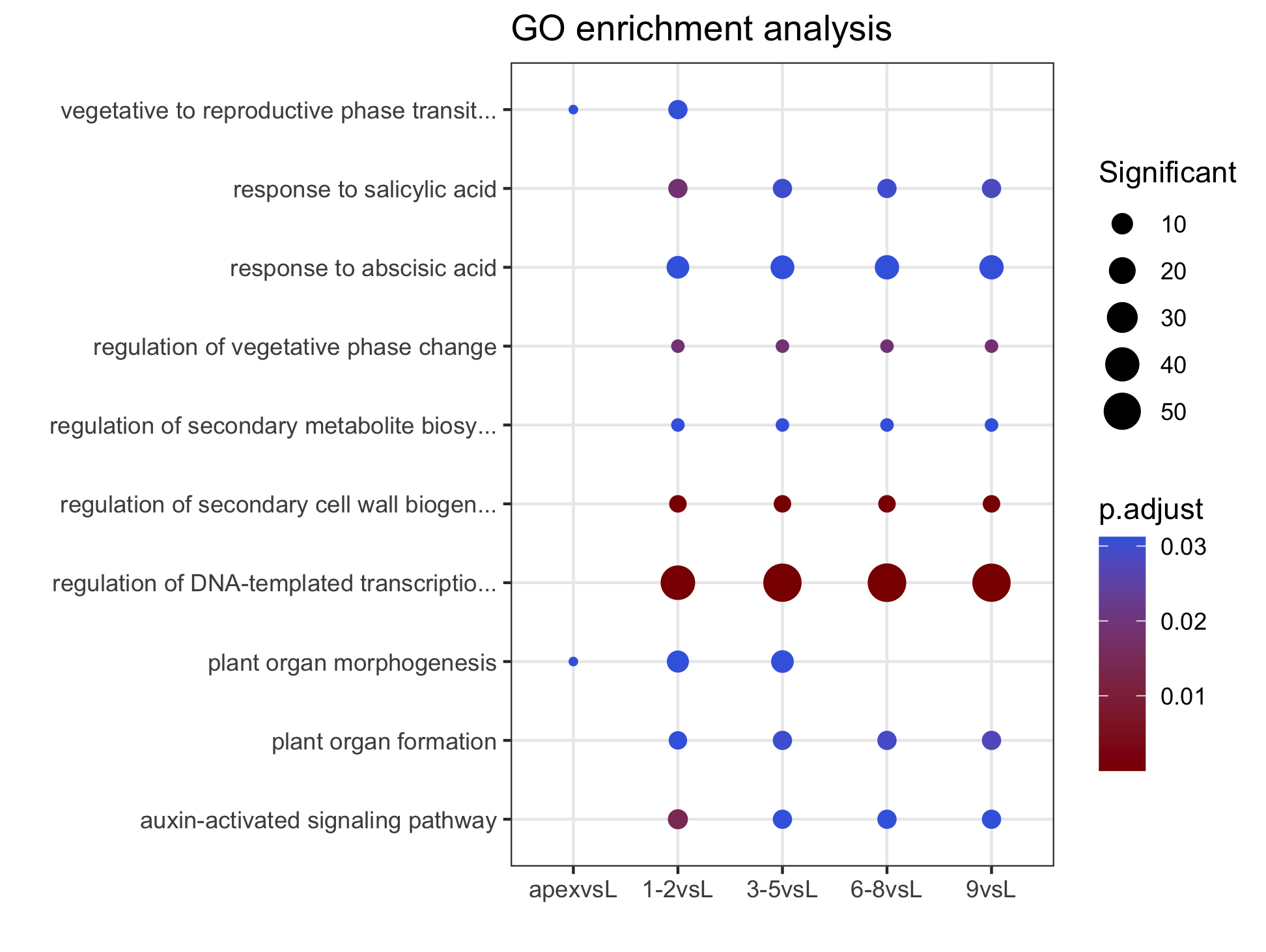

Supplement: Supplementary file 1 — Supplementary Material 1 [file 41598_2025_28485_MOESM1_ESM.zip › Supplements_B10/S12.GO_targets_enrichment_leaves.png]

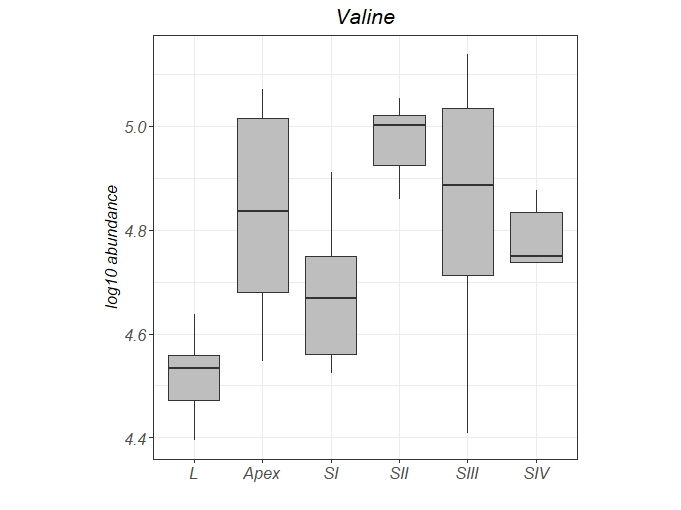

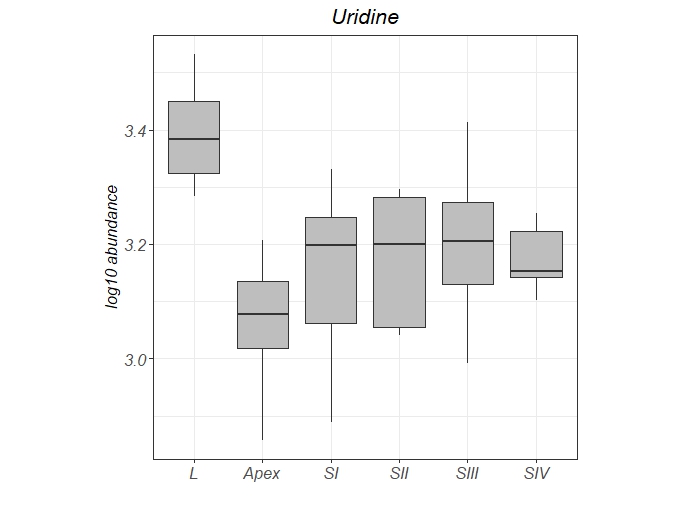

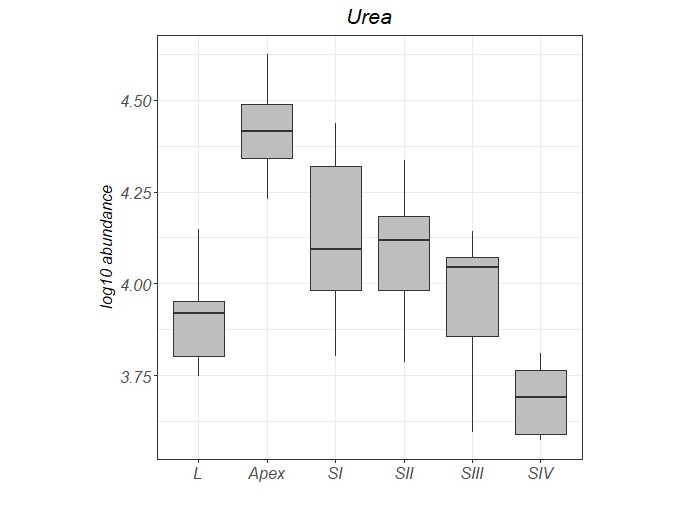

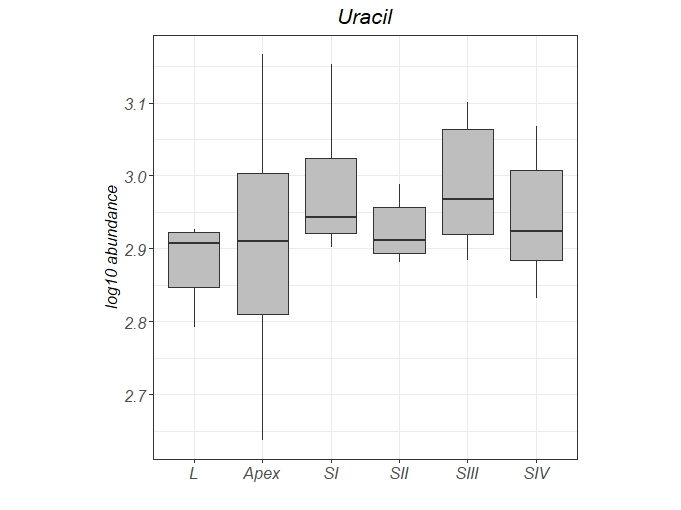

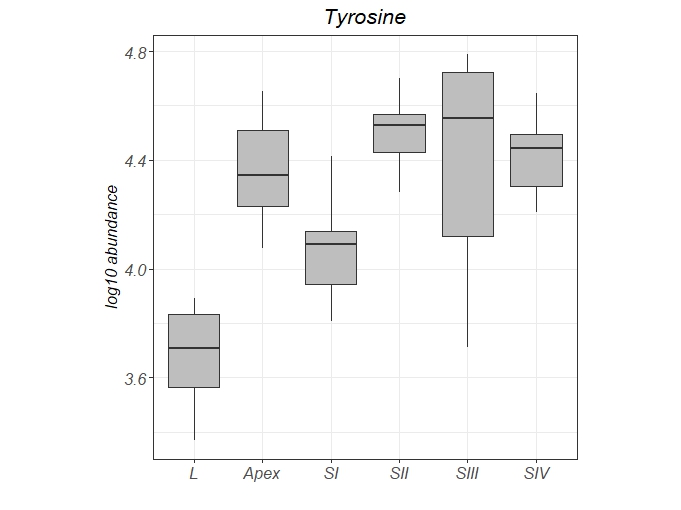

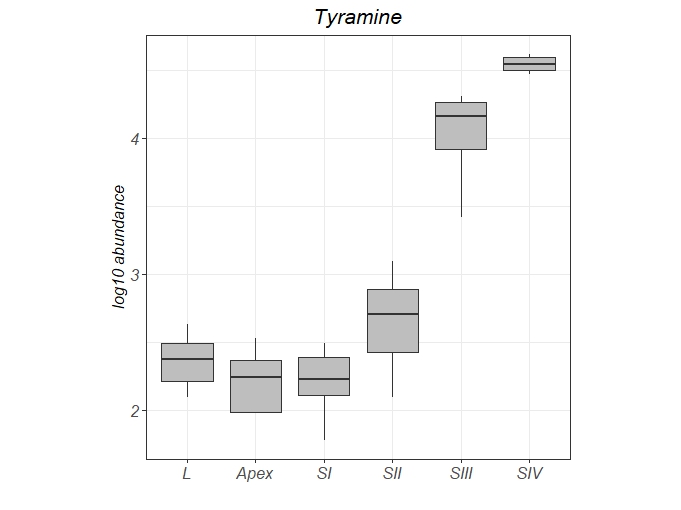

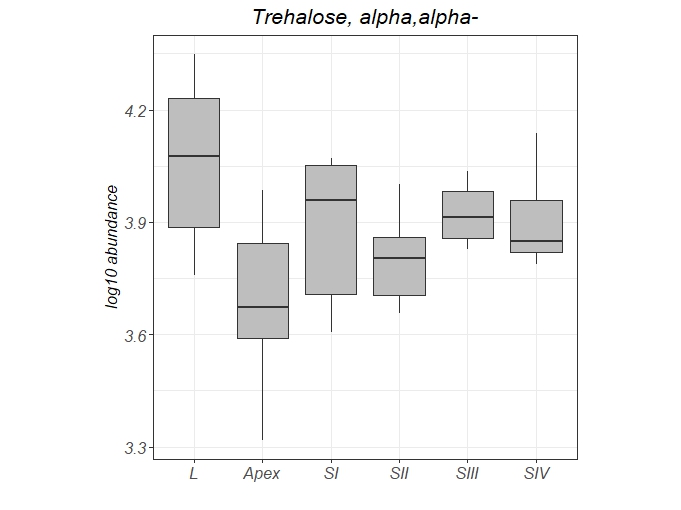

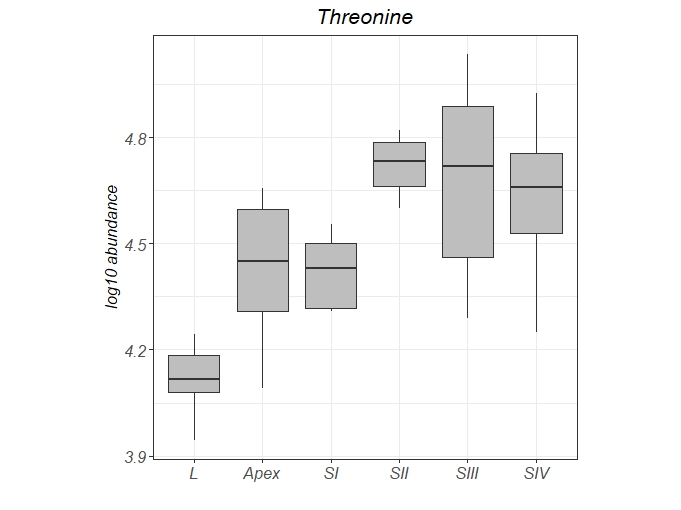

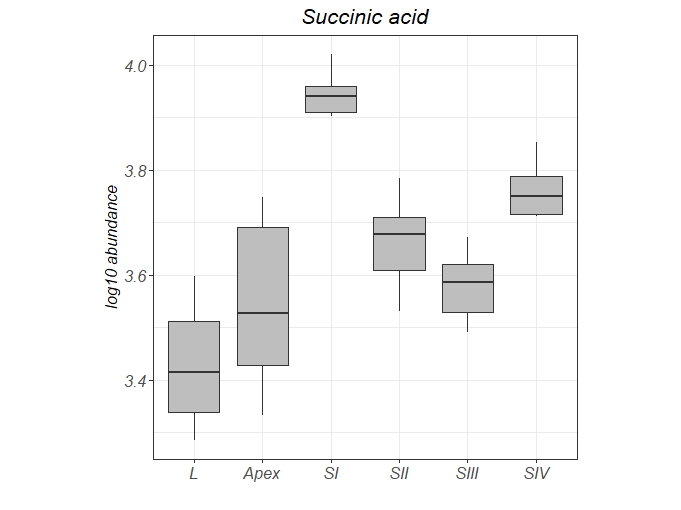

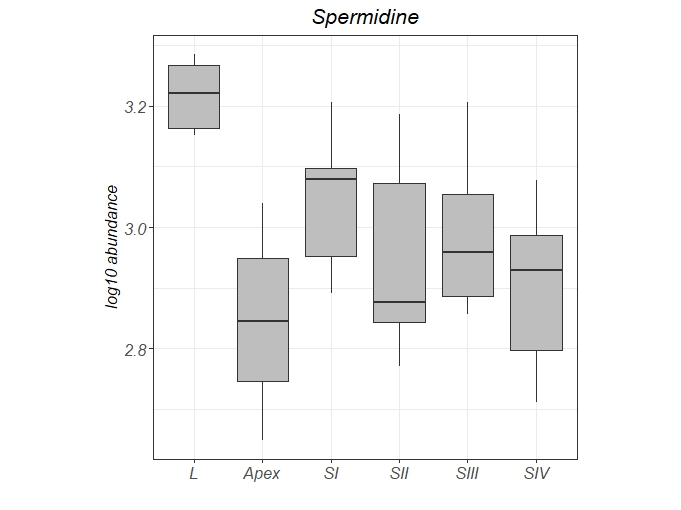

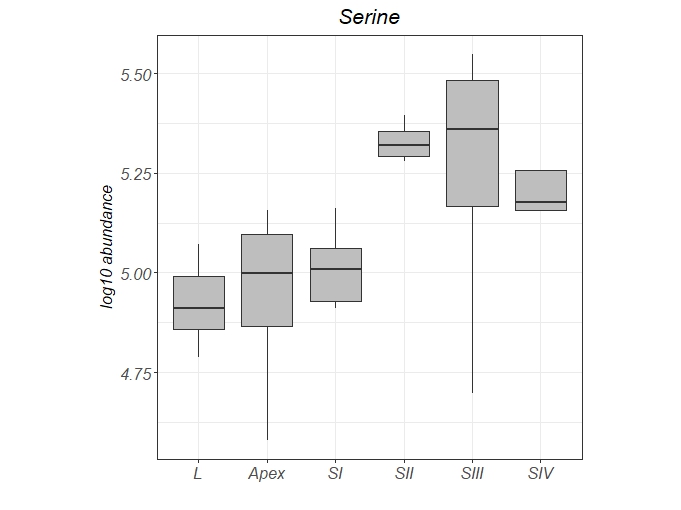

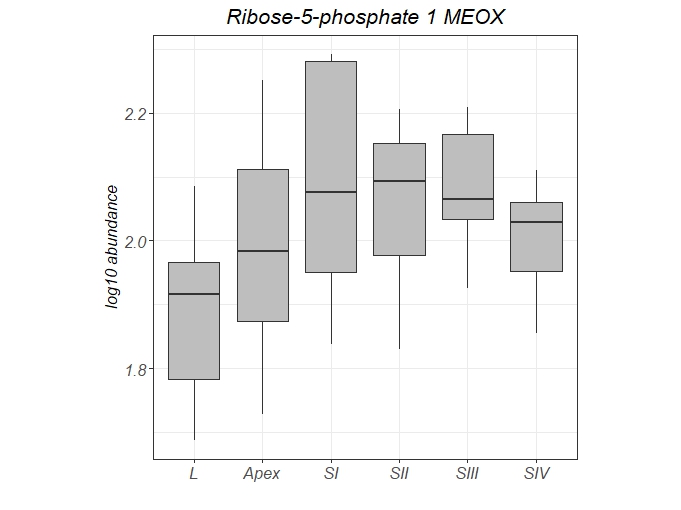

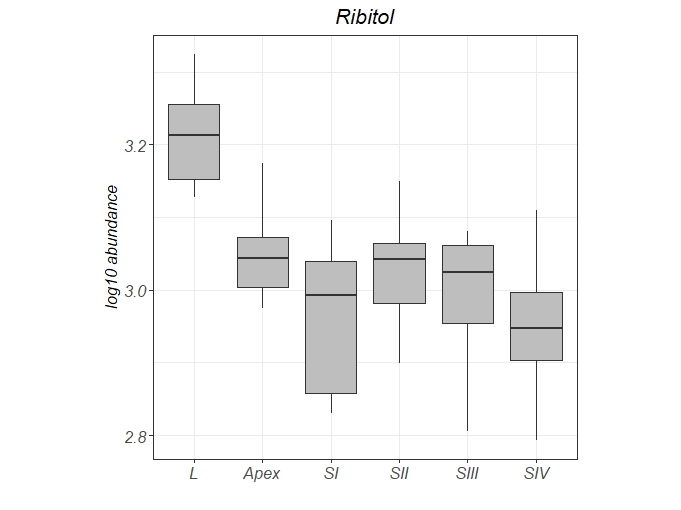

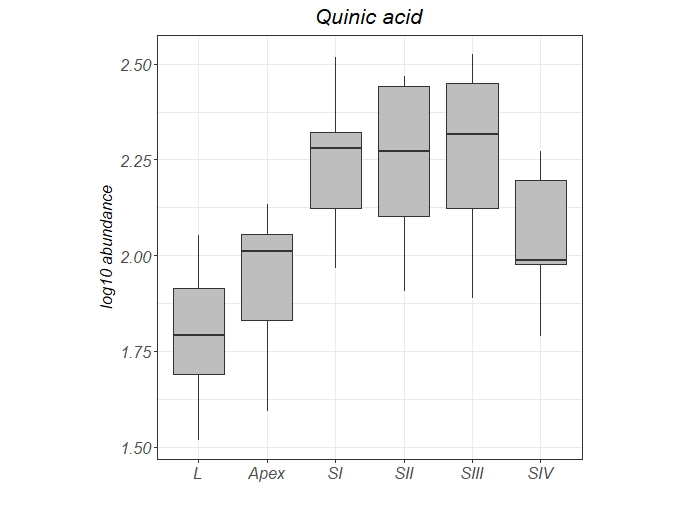

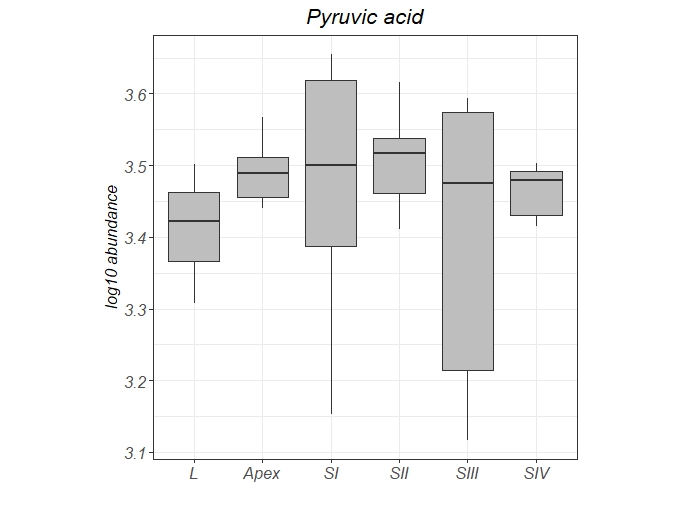

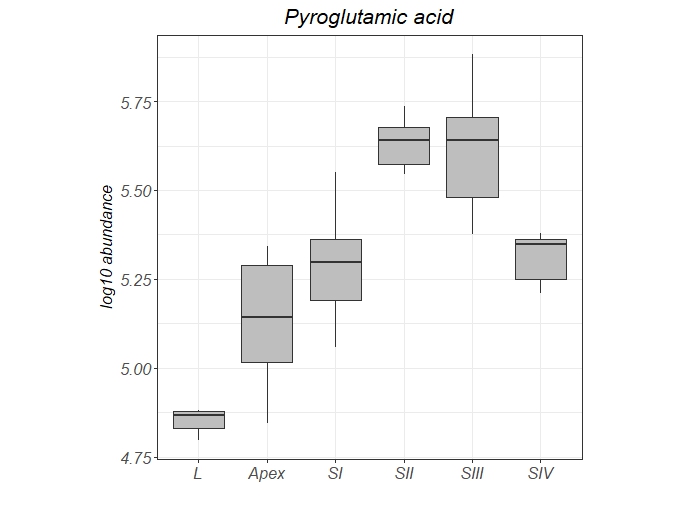

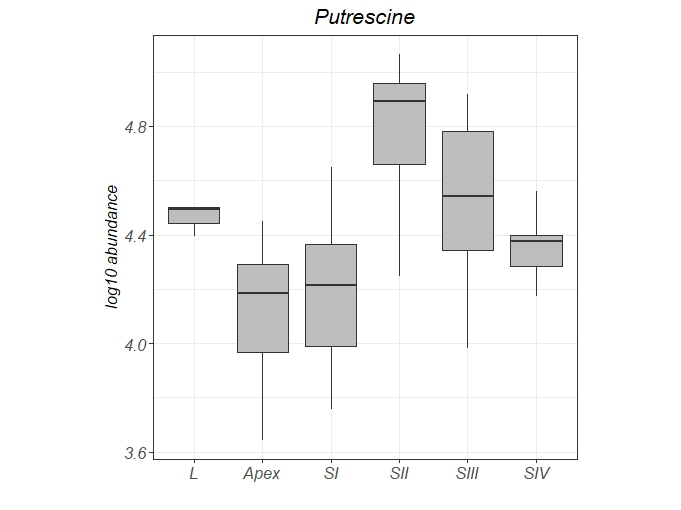

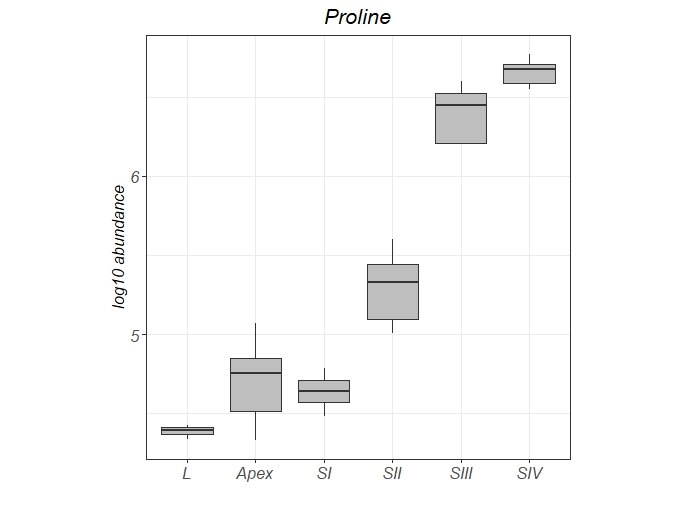

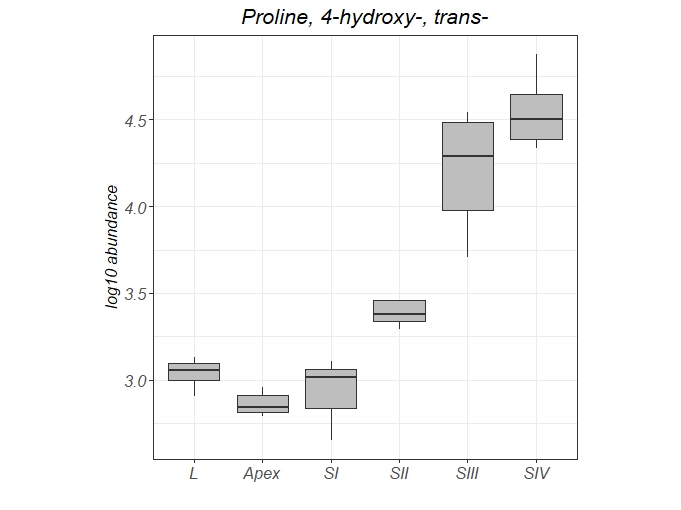

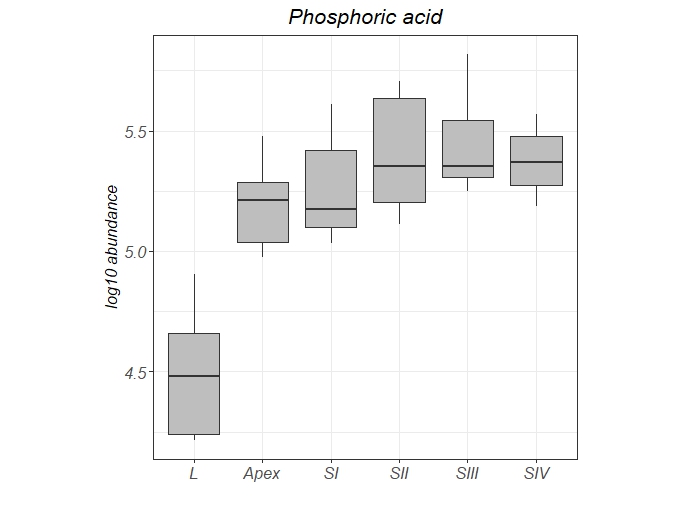

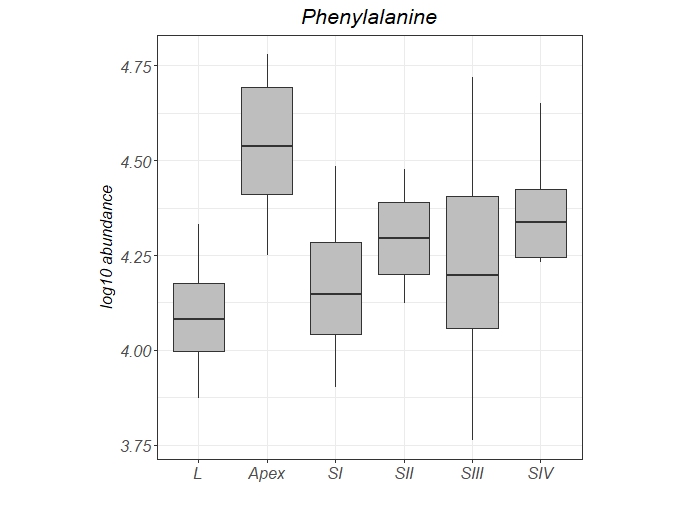

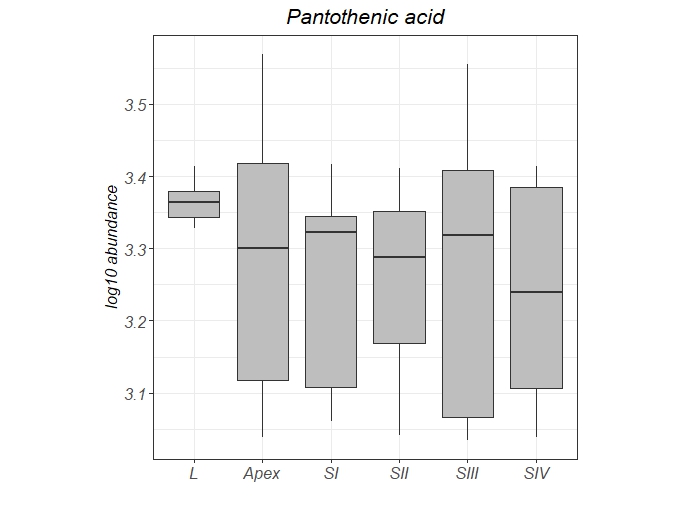

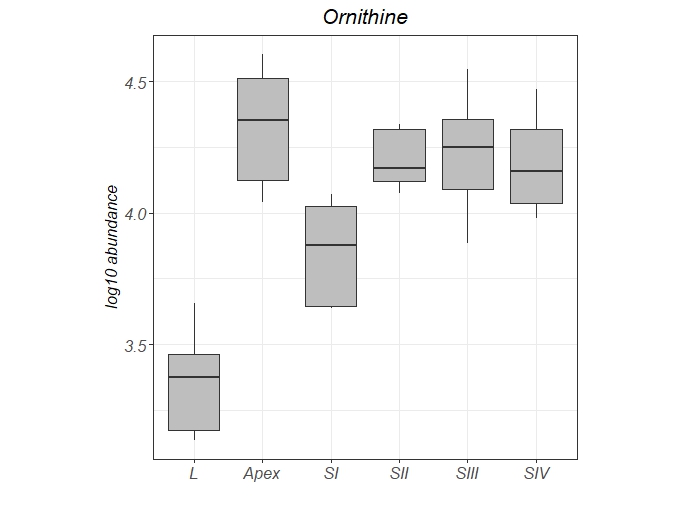

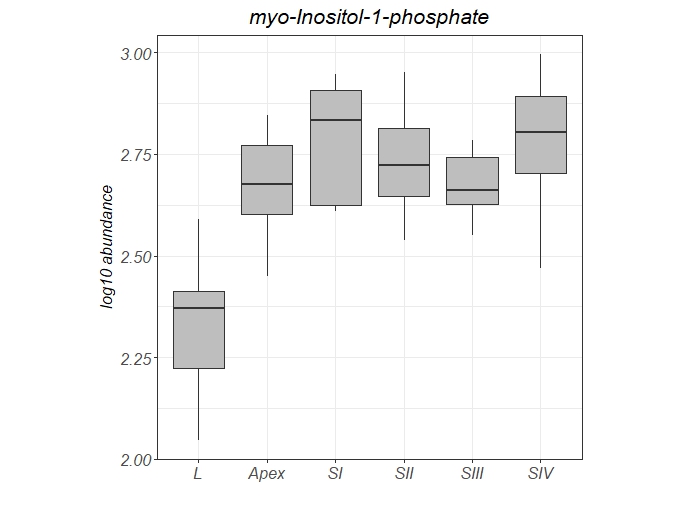

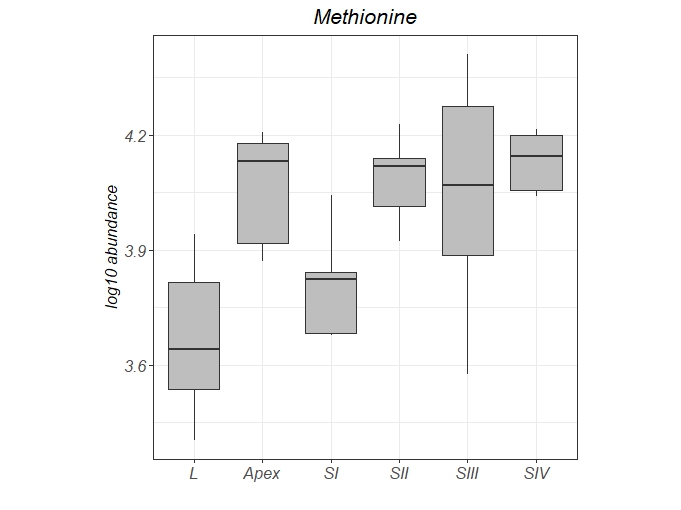

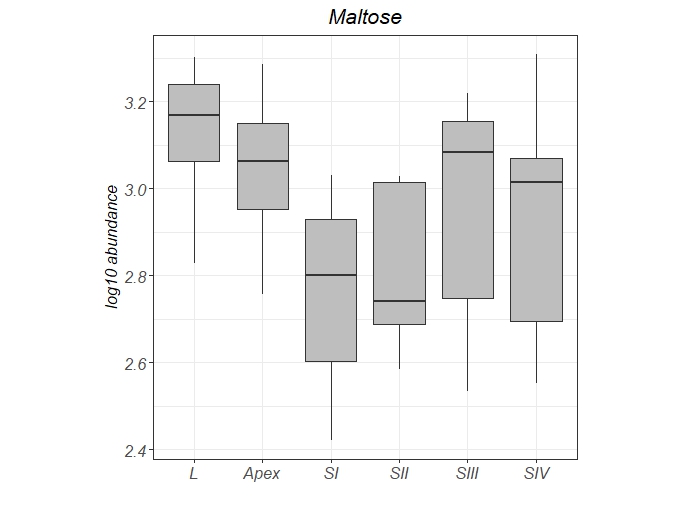

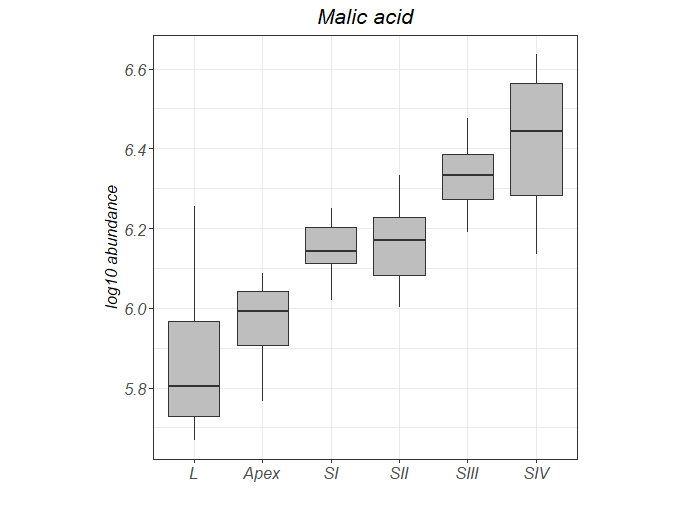

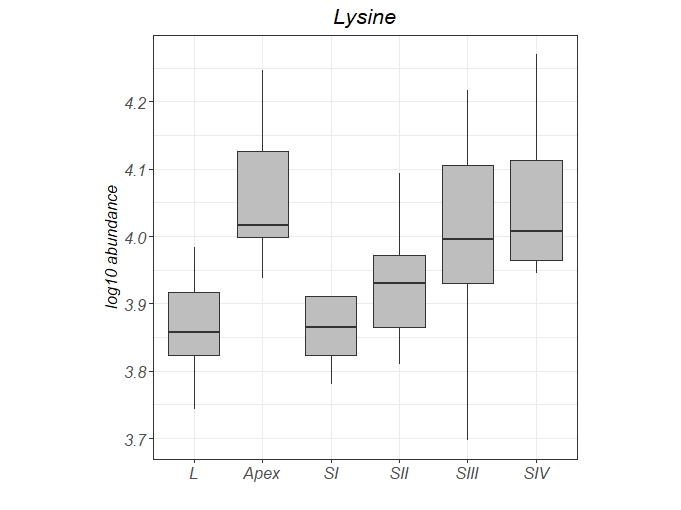

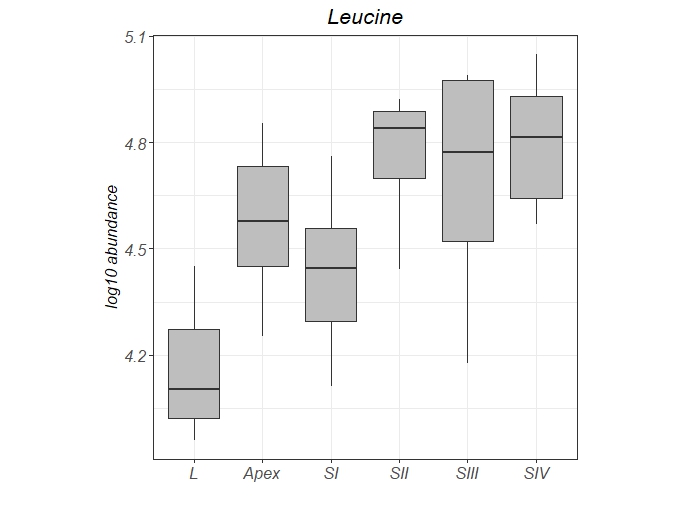

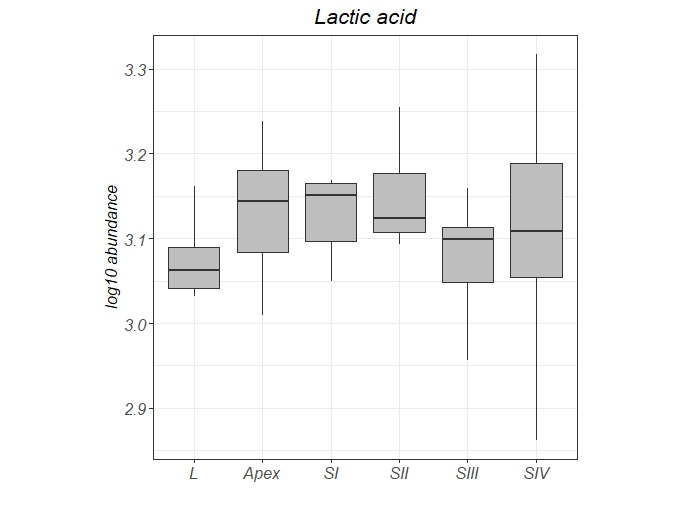

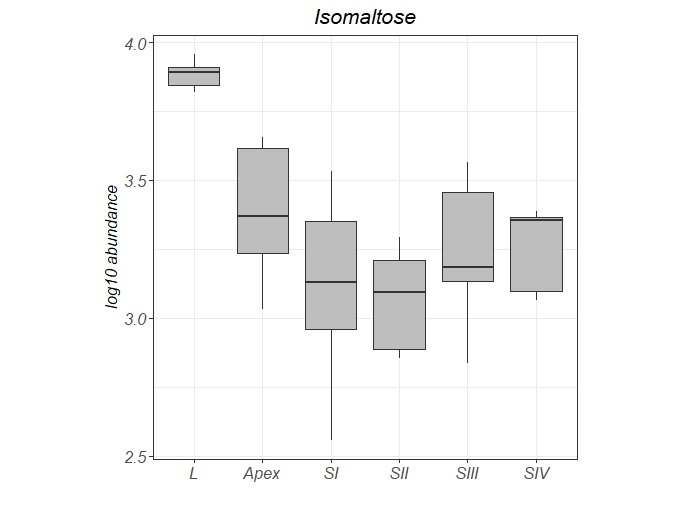

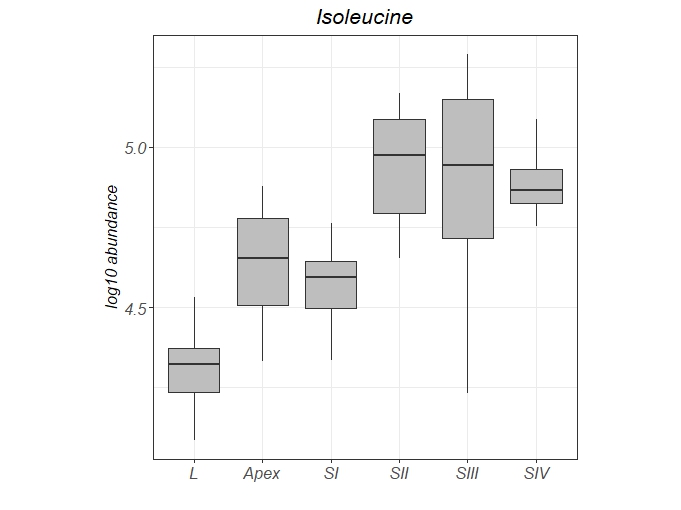

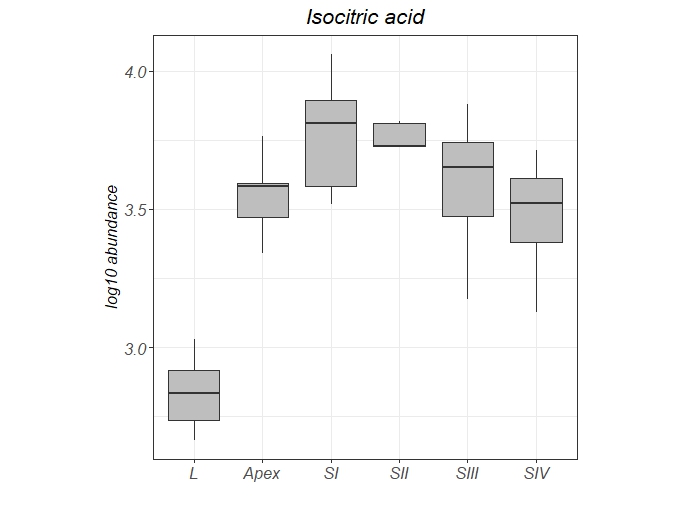

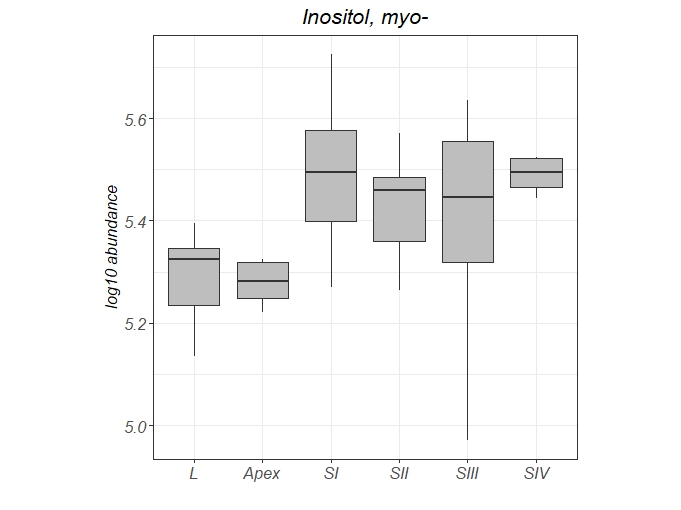

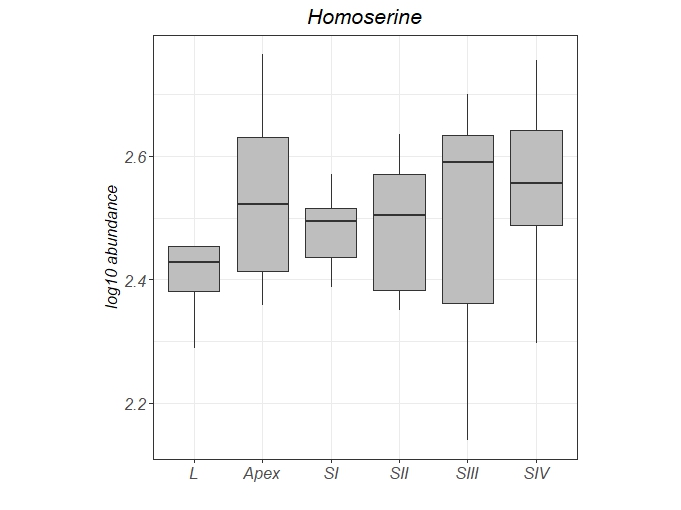

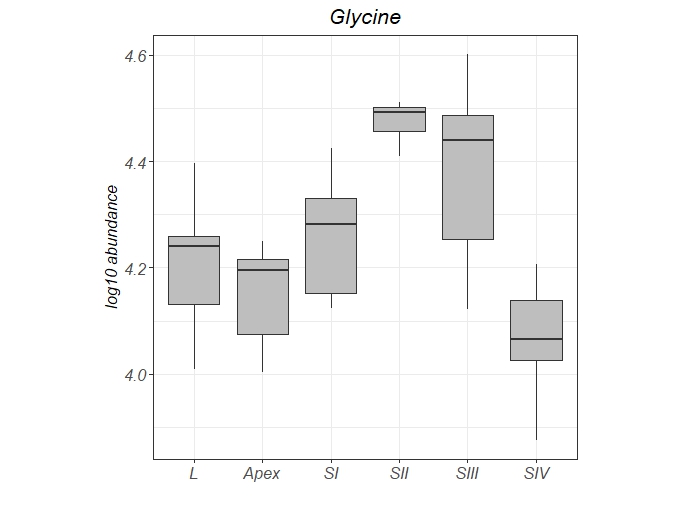

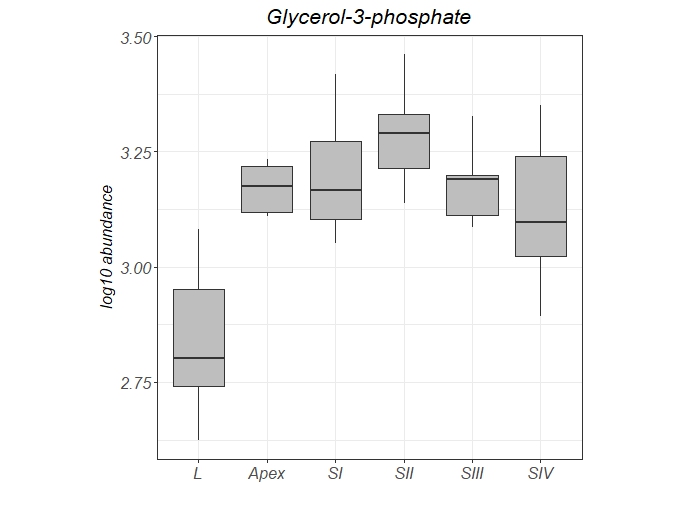

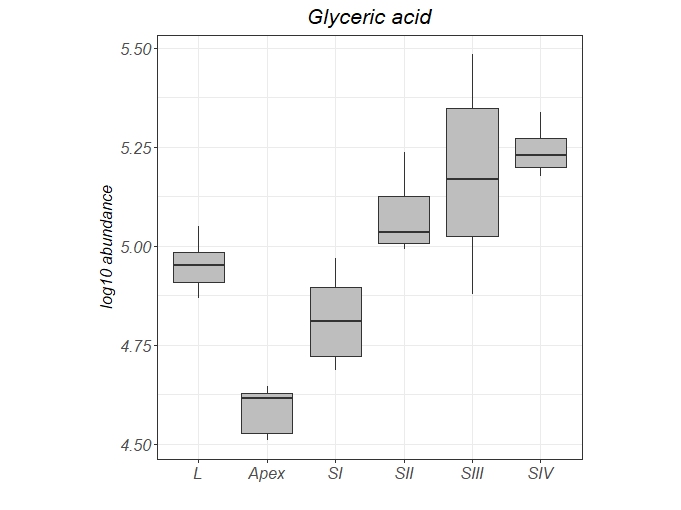

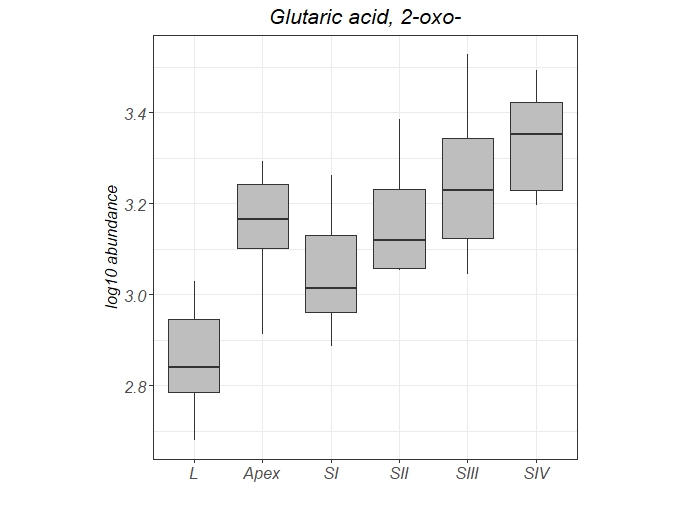

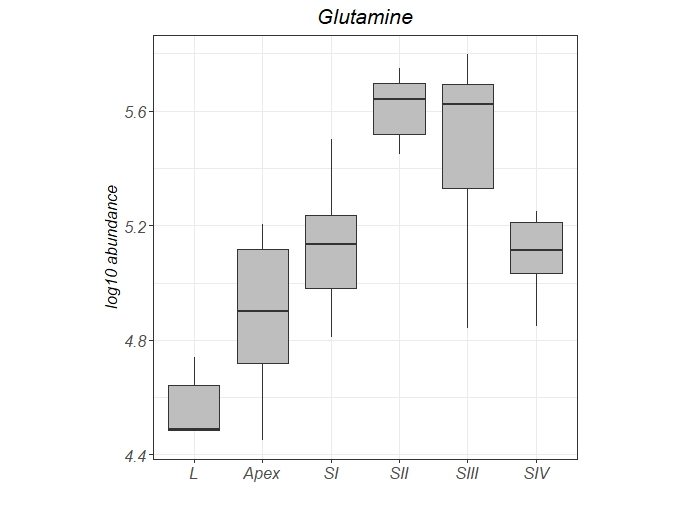

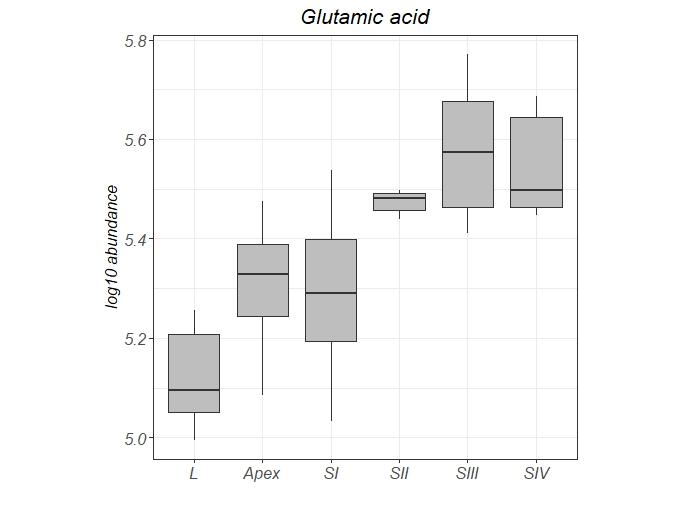

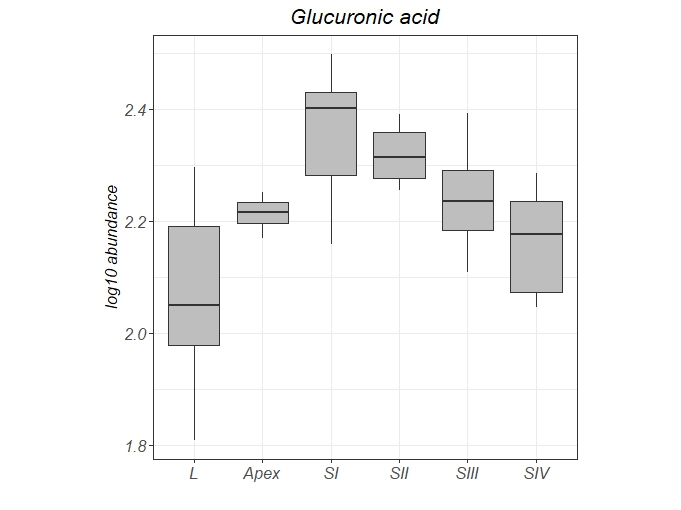

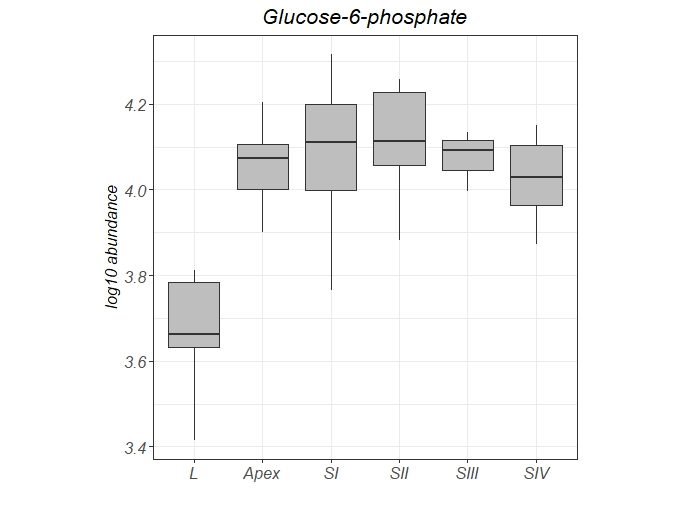

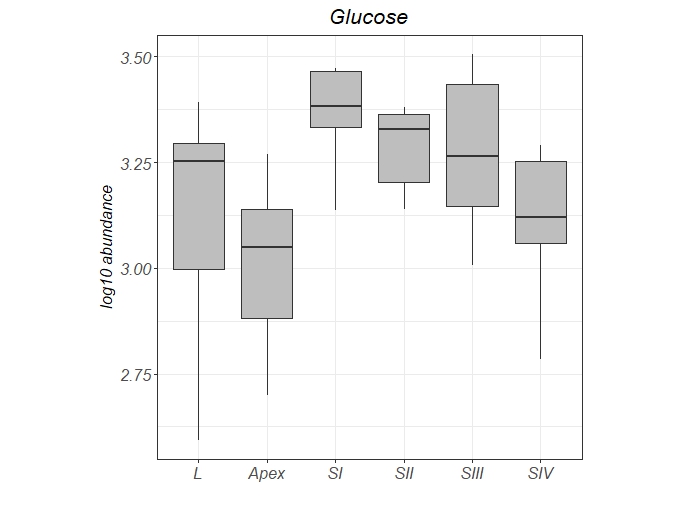

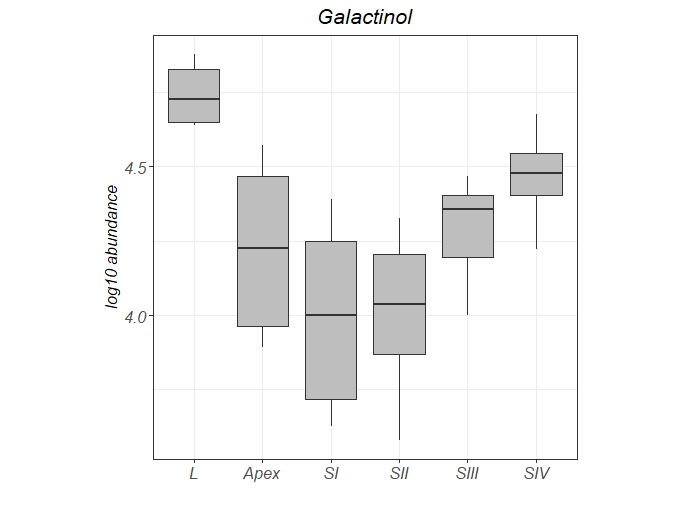

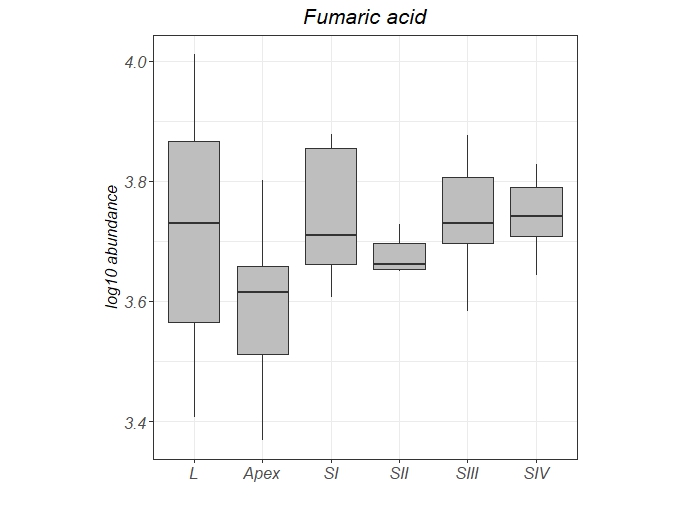

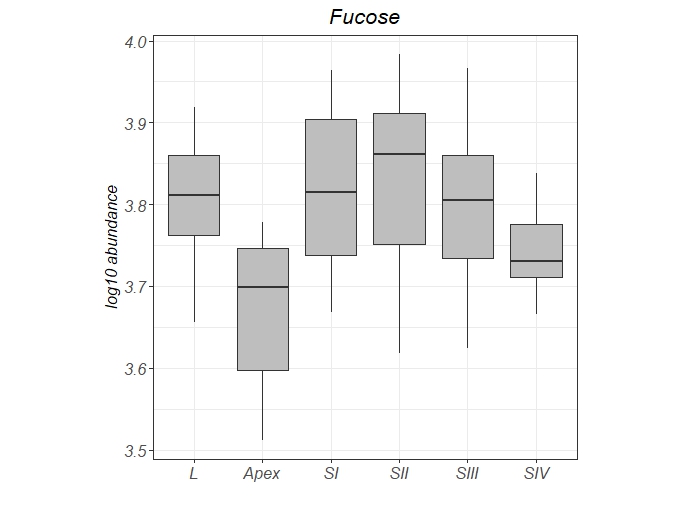

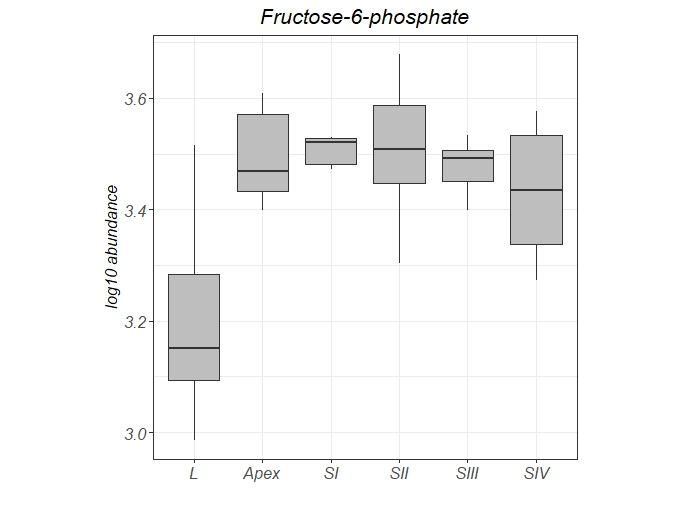

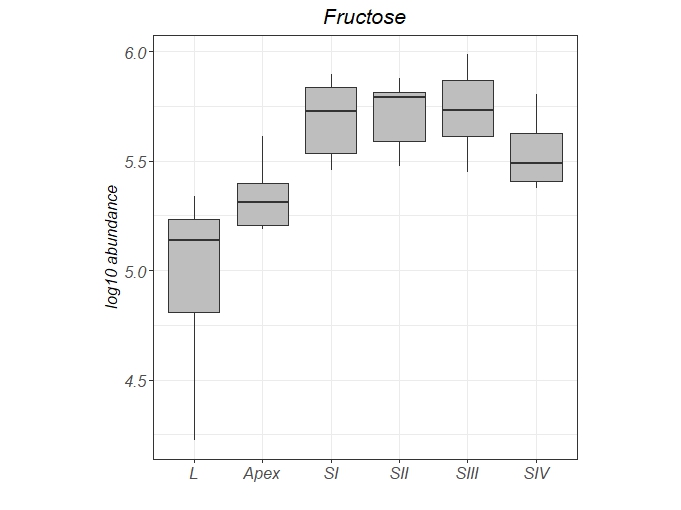

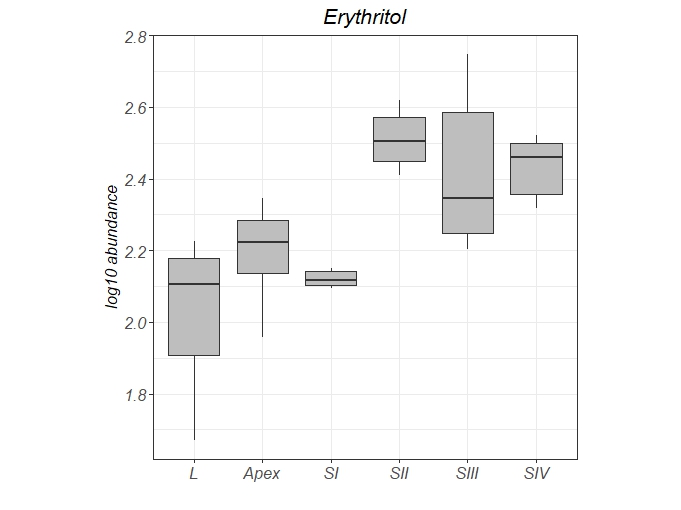

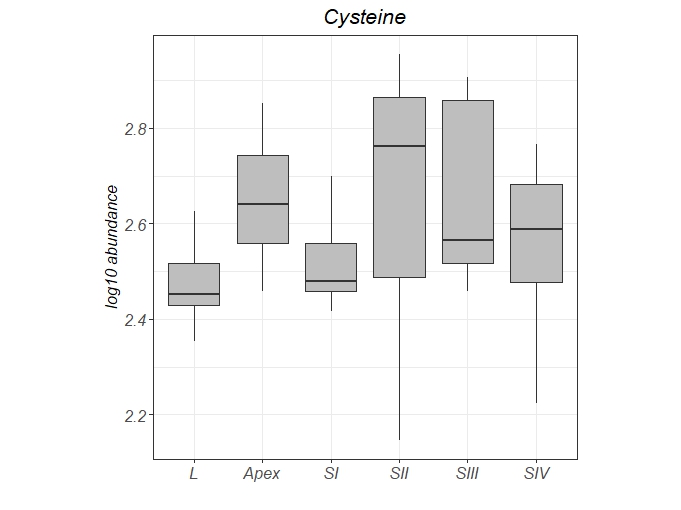

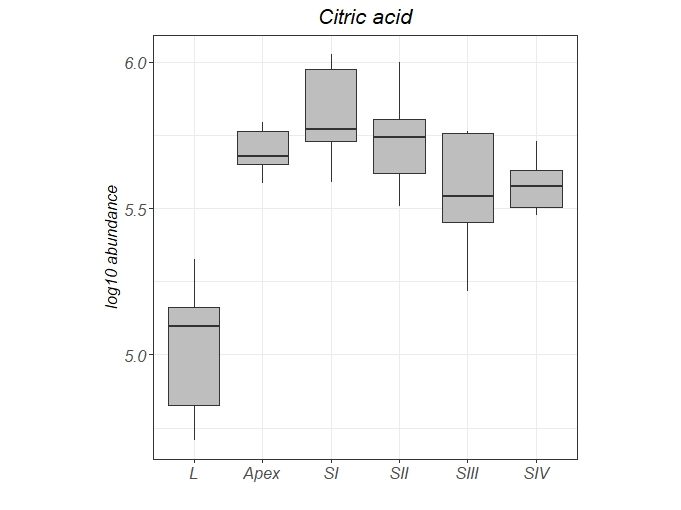

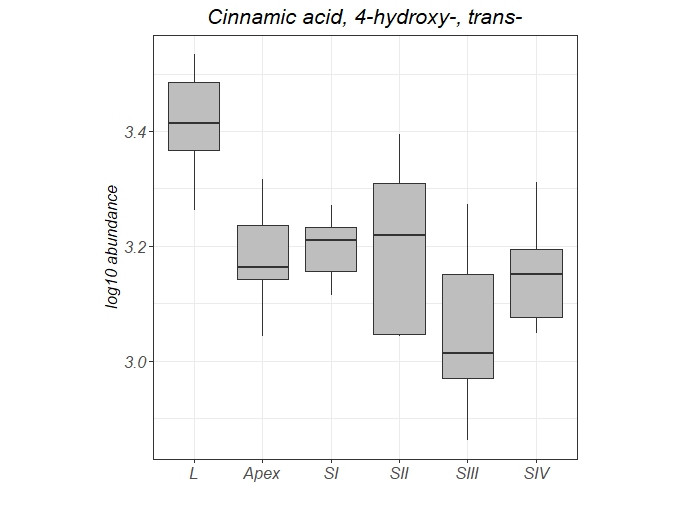

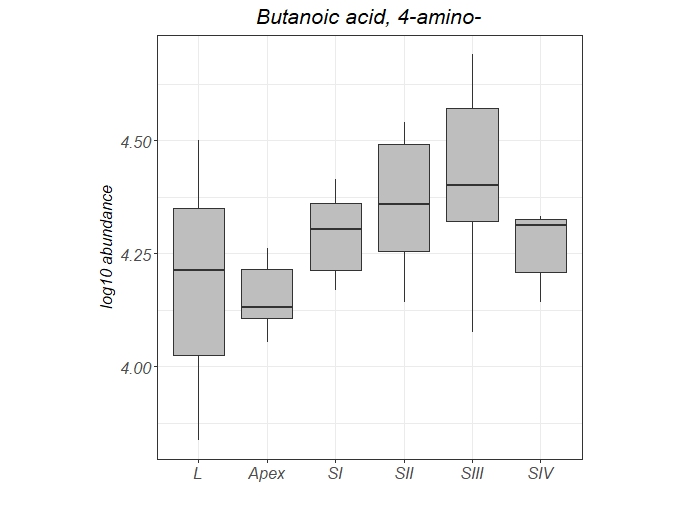

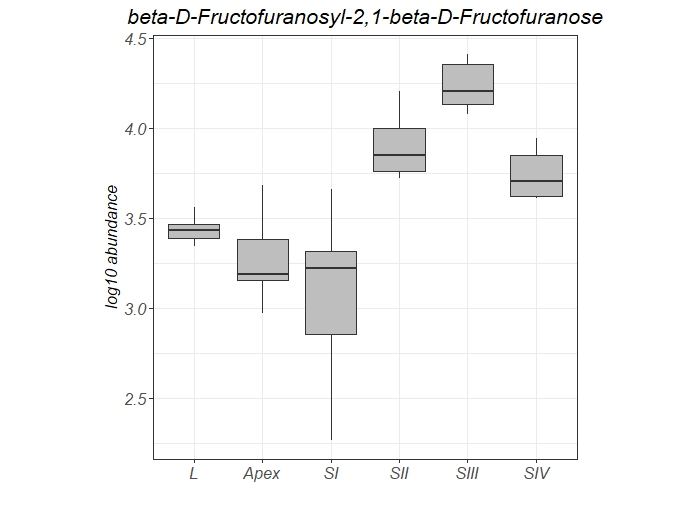

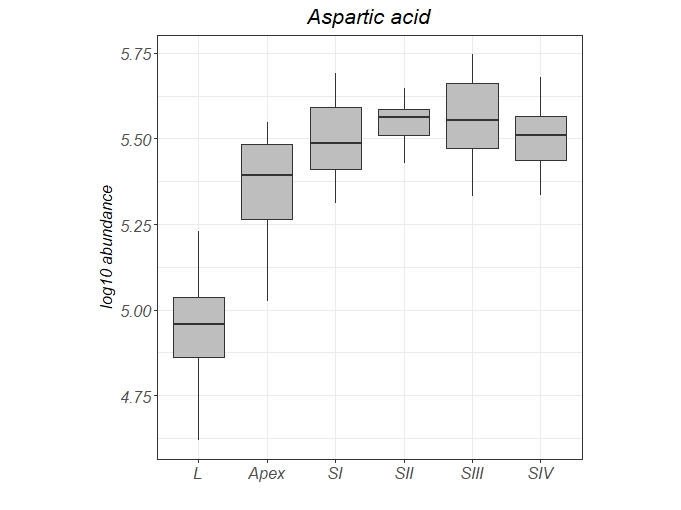

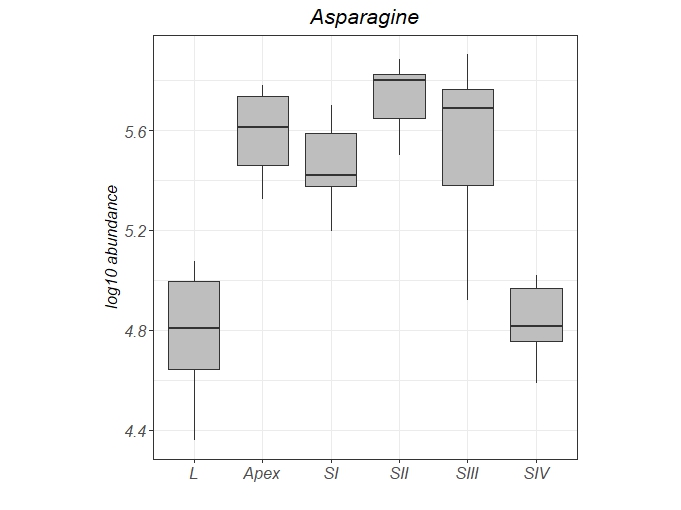

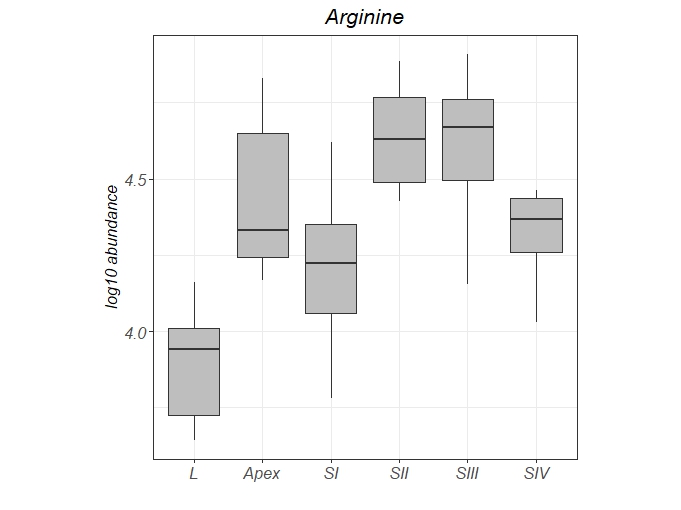

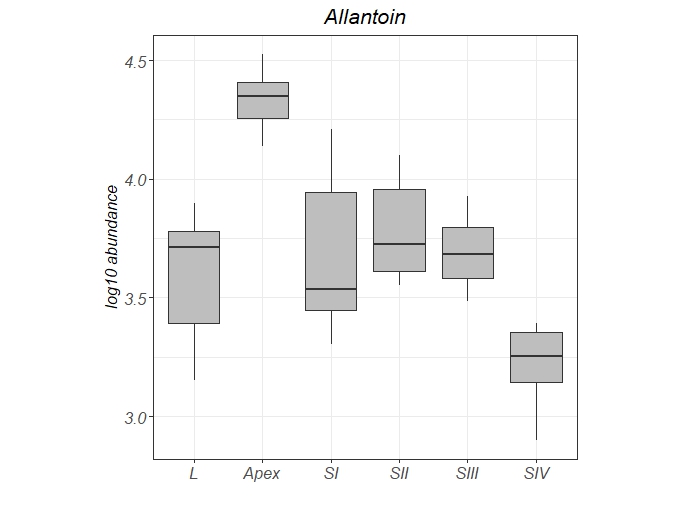

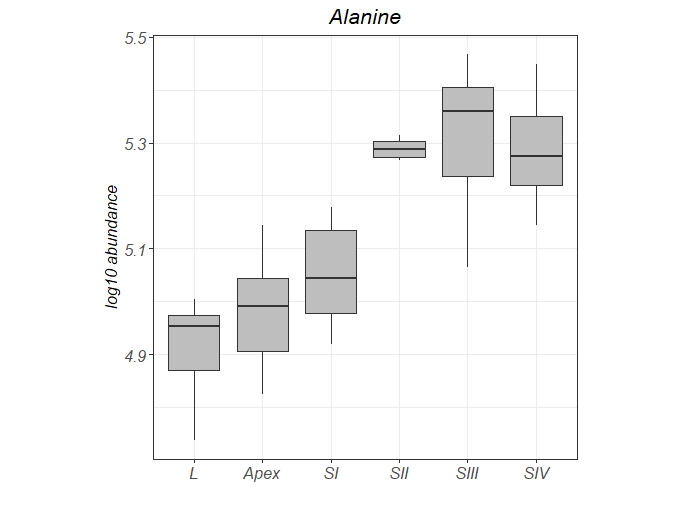

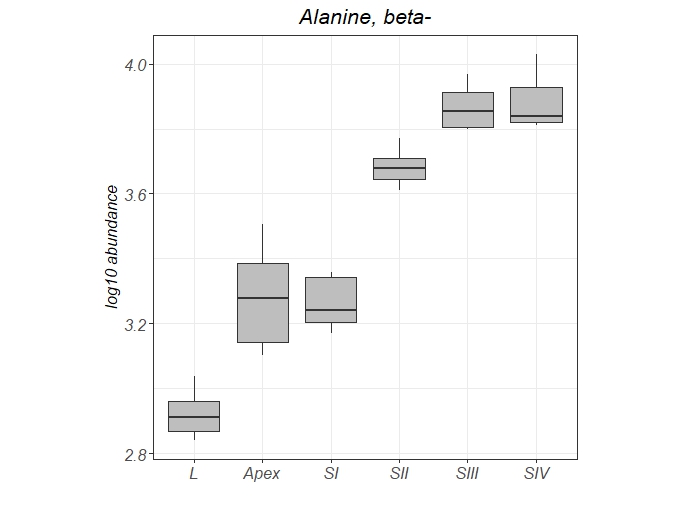

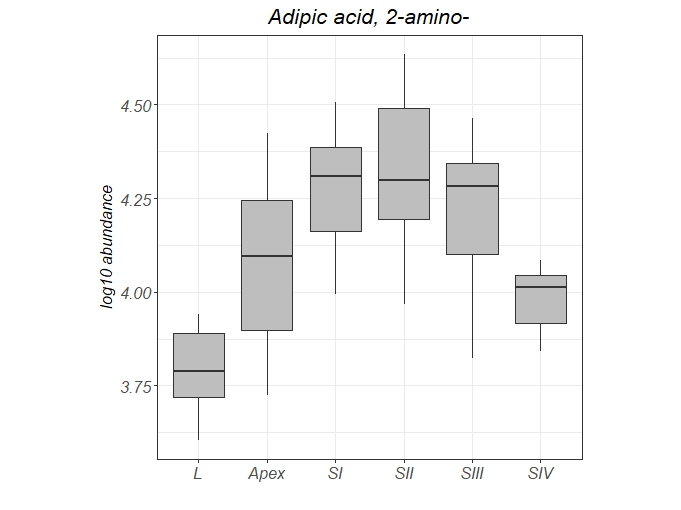

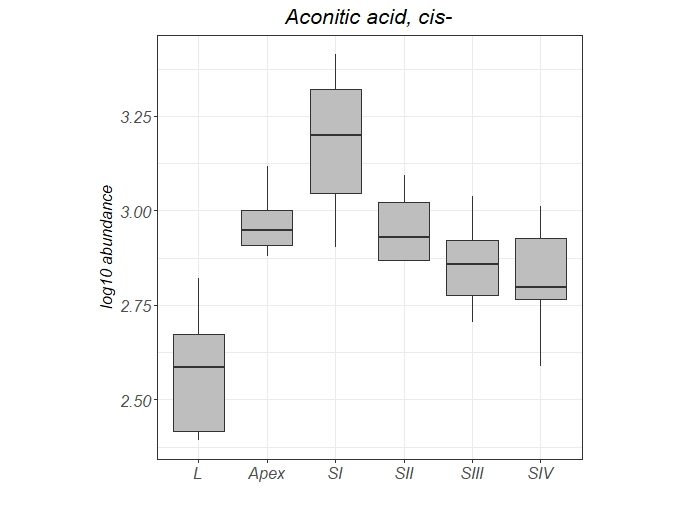

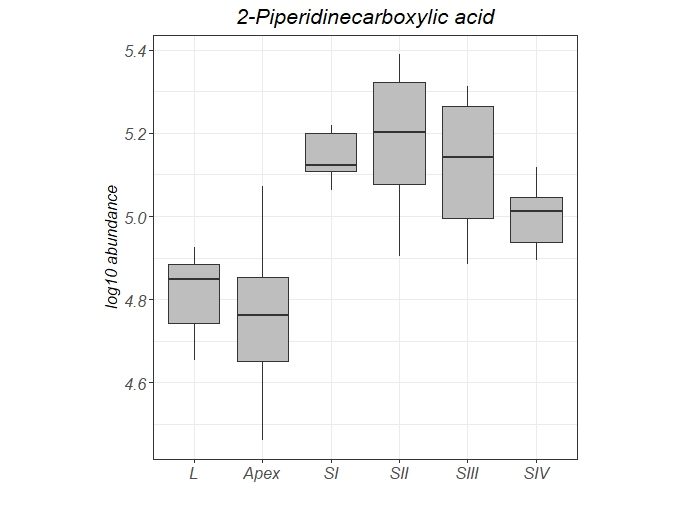

Supplement: Supplementary file 1 — Supplementary Material 1 [file 41598_2025_28485_MOESM1_ESM.zip › Supplements_B10/S17.Metabolite_boxplots.docx]

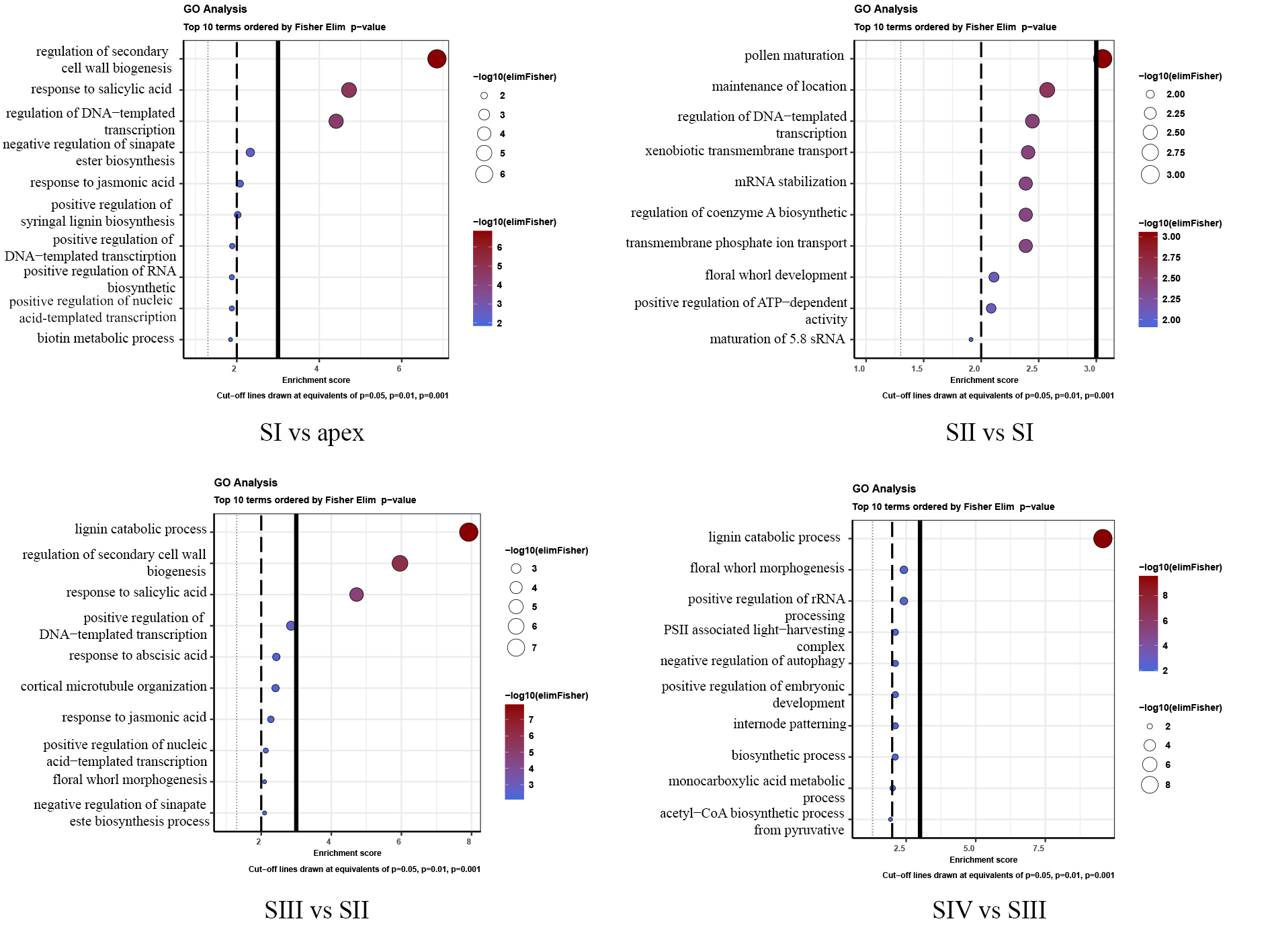

Supplement: Supplementary file 1 — Supplementary Material 1 [file 41598_2025_28485_MOESM1_ESM.zip › Supplements_B10/S11.GO_targets_enrichment_dev_stages.png]
